# Entertainment activities and the risk of Alzheimer’s disease: a Mendelian randomization analysis

**DOI:** 10.3389/fnagi.2024.1419317

**Published:** 2024-06-04

**Authors:** Tianqi Lu, Lilin Wang, Yunhua Zheng, Hua Liu, Jianyu Liu

**Affiliations:** ^1^Obesity and Metabolism Medicine-Engineering Integration Laboratory, Department of General Surgery, The Third People's Hospital of Chengdu, The Affiliated Hospital of Southwest Jiaotong University, Chengdu, China; ^2^Center of Gastrointestinal and Minimally Invasive Surgery, Department of General Surgery, The Third People's Hospital of Chengdu, Affiliated Hospital of Southwest Jiaotong University, Chengdu, China; ^3^Medical Research Center, The Third People’s Hospital of Chengdu, Affiliated Hospital of Southwest Jiaotong University, Chengdu, China; ^4^Sichuan Engineering Research Center for Biomimetic Synthesis of Natural Drugs, School of Life Science and Engineering, Southwest Jiaotong University, Chengdu, Sichuan, China; ^5^Department of Quality Evaluation and Medical Record Management, The Affiliated Hospital of Southwest Jiaotong University and The Third People's Hospital of Chengdu, Chengdu, Sichuan, China; ^6^Department of Neurology, The Affiliated Hospital of Southwest Jiaotong University and The Third People's Hospital of Chengdu, Chengdu, Sichuan, China

**Keywords:** Alzheimer’s disease, entertainment activities, Mendelian randomization, time spent using computer, causal relationship

## Abstract

**Background:**

Effective prevention is key to addressing the increasing prevalence and mortality of Alzheimer’s disease. Assessing the causal relationship between modifiable entertainment activity factors and the risk of Alzheimer’s disease is important for developing public health measures, but establishing causal relationships in epidemiological data may be challenging.

**Methods:**

This study using the two-sample Mendelian randomization analysis aimed to investigate the causal effect of entertainment activity factors on the risk of Alzheimer’s disease. Summary statistics from publicly available genome-wide association studies were used to analyze 14 modifiable entertainment activity. The inverse variance weighted random effects method as the primary analytical method to estimate causal effects was used. Additionally performed MR-Egger, weighted median and weighted model methods to assess the robustness of the results. The reliability of our findings was validated through systematic sensitivity analyses and tests for heterogeneity.

**Results:**

We found significant correlation between time spent using computer (odds ratio 0.998; 95% confidence interval 0.996–0.999; *p* = 0.013) and Alzheimer’s disease, compared to other studied entertainment activities that had no significant causal relationship with Alzheimer’s disease.

**Conclusion:**

Our findings support the hypothesis that increased computer use may reduce the risk of Alzheimer’s disease, providing potential strategic directions for the prevention of neurodegenerative diseases.

## 1 Introduction

Alzheimer’s disease (AD) stands as the predominant form of dementia, representing a neurodegenerative condition marked by gradual cognitive decline and behavioral disturbances. Recent data underscore a persistent rise in both the prevalence and mortality rates of AD (Alzheimer's Association, 2016). In light of the lack of pharmacological interventions capable of arresting disease progression, the emphasis is squarely on preventative strategies as pivotal in addressing AD. Further elucidation of the etiology of AD and the proposition of actionable prevention measures hold significant importance for AD prevention (Lane et al., 2018).

The cause of AD remains uncertain and is mainly related to genetics, age, environmental factors and lifestyle (Lane et al., 2018; Scheltens et al., 2021). There are challenges to formulating prevention strategies based on these risk factors, such as the fact that genetic factors are difficult to change and age is not reversible. Modifiable risk factors for diseases are those that can lower the risk of developing a specific disease through lifestyle changes or preventive measures. These factors typically stem from individual lifestyle habits, environmental influences, or health management practices, and can be easily adopted by individuals in their daily lives (Kivipelto et al., 2018). Multiple aspects have been identified as potential modifiable risk factors for AD, including diet, exercise, cognitive reserve, sleep patterns, air quality, and entertainment activities. However, the causal relationship is not yet fully understood (Livingston et al., 2017).

Investigating the causal relationship between entertainment activities as part of an individual’s adaptive behavior and the risk of AD is crucial for developing effective public health interventions. However, establishing causal relationships in epidemiological data can be challenging. Adaptive behavior encompasses a range of social, conceptual, and practical skills exhibited in daily behavior, reflecting individuals’ ability to adapt to environmental demands. It serves as a fundamental basis for assessing intellectual function (Tassé et al., 2012). In modern lifestyles, entertainment activities occupy a significant portion of adaptive behavior. Therefore, focusing on entertainment activities within daily adaptive behaviors may be a meaningful strategy for AD prevention. Entertainment activities typically encompass a range of pursuits that individuals engage in for relaxation, pleasure, or excitement, often characterized by their practical applicability. Previous research has demonstrated that serious games can enhance the quality of life for older adults with cognitive impairment by improving learning function and promoting physical activity (Abd-Alrazaq et al., 2023). Additionally, exercise games or training observed in patients with AD have been shown to support healthy aging and exert neuroprotective effects on the brain (Stanmore et al., 2017; Huuha et al., 2022). However, the number of epidemiological studies exploring the causal association between entertainment activities and AD remains relatively limited (Kuźma et al., 2018). Furthermore, these studies often focus on specific types of activities or populations and have not yet established a comprehensive and systematic research framework. Research on the effects of entertainment activities on AD is still in its early stages, and many findings require further validation and in-depth exploration (Ströhle et al., 2015; Stanmore et al., 2017; Abd-Alrazaq et al., 2023).

The application of traditional epidemiological studies to evaluate the causal relationship between adaptive behavior factors and the risk of AD is limited by confounding factors and potential bias from reverse causation (Davies et al., 2018). Mendelian randomization (MR) offers a methodological approach within epidemiology that uses genetic variants as instrumental variables (IV) to infer causality between risk factors and the outcome of interest. MR provides unbiased estimates of causality because genetic variants are inherited randomly from parents to offspring, independent of potential confounders. Therefore, this analytical method yields more definitive evidence regarding causality (Bowden and Holmes, 2019; Richmond and Davey, 2022).

The objective of this study is to investigate the influence of entertainment activities on AD within individual lifestyles. Here, we conducted a two-sample MR analysis to evaluate the causal impact of 14 entertainment activity factors on the risk of AD. The findings from this study could offer significant insights for preventing AD and contribute to the development of relevant prevention strategies.

## 2 Materials and methods

### 2.1 Study design and data sources

This two-sample MR analysis was performed following the instructions of the STROBE-MR checklist. Sensitivity analyses and single nucleotide polymorphism (SNP) filtering were performed according to the guidelines (Sanderson et al., 2022). Using summary statistics from the publicly available genome-wide association studies (GWAS) on the following 14 entertainment activities: Leisure/social activities: Adult education class (Adult education), Leisure/social activities: Pub or social club (Pub/club), Leisure/social activities: Religious group (Religious group), Leisure/social activities: Sports club or gym (Sports/gym), Leisure/social activities: Other group activity (Leisure(Other group)), Leisure/social activities: None of the above (Leisure(None)), Number of days/week walked 10+ minutes (Physical(light)), Number of days/week of moderate physical activity 10+ minutes (Physical(moderate)), Number of days/week of vigorous physical activity 10+ minutes (Physical(vigorous)), Time spent driving (Driving), Time spent outdoors in summer (Outdoors(summer)), Time spent outdoors in winter (Outdoors(winter)), Time spent using computer (Computer), Time spent watching television (TV)(TV); outcome: Alzheimer’s disease (AD). Included study sample sizes ranged from 310,555 to 488,285 individuals, all of European ancestry. Details of traits and corresponding studies are provided in Table 1.

**Table 1:** Summary of the genome-wide association studies (GWAS) included in this two-sample MR study.

Category	Exposures/outcomes	Dataset	Sample size	Number of SNPs	Population	First author/Year
Alzheimer’s disease	Alzheimer’s diseases	ieu-b-5067	488,285	12,321,875	European	Woolf B/2022
Adaptive behavior	Leisure/social activities: Adult education class	ukb-b-1553	461,369	9,851,867	European	Elsworth B/2018
Adaptive behavior	Leisure/social activities: Pub or social club	ukb-b-4171	461,369	9,851,867	European	Elsworth B/2018
Adaptive behavior	Leisure/social activities: Religious group	ukb-b-4667	461,369	9,851,867	European	Elsworth B/2018
Adaptive behavior	Leisure/social activities: Sports club or gym	ukb-b-4000	461,369	9,851,867	European	Elsworth B/2018
Adaptive behavior	Leisure/social activities: Other group activity	ukb-b-5076	461,369	9,851,867	European	Elsworth B/2018
Adaptive behavior	Leisure/social activities: None of the above	ukb-b-4077	461,369	9,851,867	European	Elsworth B/2018
Adaptive behavior	Time spent doing light physical activity	ukb-b-4886	454,783	9,851,867	European	Elsworth B/2018
Adaptive behavior	Time spent doing moderate physical activity	ukb-b-4710	440,266	9,851,867	European	Elsworth B/2018
Adaptive behavior	Time spent doing vigorous physical activity	ukb-b-151	440,512	9,851,867	European	Elsworth B/2018
Adaptive behavior	Time spent driving	ukb-b-3793	310,555	9,851,867	European	Elsworth B/2018
Adaptive behavior	Time spent outdoors in summer	ukb-b-969	419,314	9,851,867	European	Elsworth B/2018
Adaptive behavior	Time spent outdoors in winter	ukb-b-6811	364,465	9,851,867	European	Elsworth B/2018
Adaptive behavior	Time spent using computer	ukb-b-4522	360,895	9,851,867	European	Elsworth B/2018
Adaptive behavior	Time spent watching television (TV)	ukb-b-5192	437,887	9,851,867	European	Elsworth B/2018

### 2.2 Instrumental variable selection

For each exposure included in this analysis, we selected genetic instruments that were statistically significant at a threshold of *p* < 5 × 10^−8^ based on the published GWAS for the respective trait, utilizing publicly available summary statistics. Subsequently, we conducted linkage disequilibrium (LD) clumping to ensure independence among the instruments used for each trait. This was achieved by selecting only the SNP with the lowest *p*-value from all SNPs exhibiting an LD r^2^ ≥ 0.001. To minimize the impact of weak instrument bias on causal inference, the F-statistic values were all above 10. And applied the Steiger filter to remove SNPs with a larger R-Squared in AD than entertainment activities (Supplementary Table S1).

The GWAS data for Leisure/social activities was obtained from the UK Biobank, which conducted a survey on Leisure/social activities among 461,369 individuals. Classify the response to the question “Which of the following do you attend once a week or more often? (You can select more than one)” into six distinct categories: “Adult education class” (ukb-b-1553), “Pub or social club” (ukb-b-4171), “Religious group” (ukb-b-4667), “Sports club or gym” (ukb-b-4000), “Other group activity” (ukb-b-5076) and “None of the above” (ukb-b-4077). The instrumental variables employed in this study encompassed the following categories: Adult education (4 SNPs), Pub/club (18 SNPs), Religion group (23 SNPs), Sports/Gym (7 SNPs), Leisure (other group) (4 SNPs), and Leisure (None) (10 SNPs).

The GWAS data for Physical (light) was obtained from the UK Biobank (ukb-b-4886), which conducted a survey on Physical(light) among 454,783 individuals. Classify the response to the question “In a typical WEEK, on how many days did you walk for at least 10 min at a time? (Include walking that you do at work, traveling to and from work, and for sport or leisure).” A total of 14 SNPs as instruments from Physical(light).

The GWAS data for Physical(moderate) was obtained from the UK Biobank (ukb-b-4710), which conducted a survey on Physical(moderate) among 440,266 individuals. Classify the response to the question “In a typical WEEK, on how many days did you do 10 min or more of moderate physical activities like carrying light loads, cycling at a normal pace? (Do not include walking).” A total of 15 SNPs as instruments from Physical(moderate).

The GWAS data for Physical(vigorous) was obtained from the UK Biobank (ukb-b-151), which conducted a survey on Physical(vigorous) among 440,512 individuals. Classify the response to the question “In a typical WEEK, how many days did you do 10 min or more of vigorous physical activity? (These are activities that make you sweat or breathe hard such as fast cycling, aerobics, heavy lifting).” A total of 9 SNPs as instruments from Physical(vigorous).

The GWAS data for Driving was obtained from the UK Biobank (ukb-b-3793), which conducted a survey on Driving among 310,555 individuals. Classify the response to the question “In a typical DAY, how many hours do you spend driving?” A total of 6 SNPs as instruments from Driving.

The GWAS data for Outdoors(summer) was obtained from the UK Biobank (ukb-b-969), which conducted a survey on Outdoors(summer) among 419,314 individuals. Classify the response to the question “In a typical DAY in summer, how many hours do you spend outdoors?” A total of 39 SNPs as instruments from Outdoors(summer).

The GWAS data for Outdoors(winter) was obtained from the UK Biobank (ukb-b-6811), which conducted a survey on Outdoors(winter) among 364,465 individuals. Classify the response to the question “In a typical DAY in winter, how many hours do you spend outdoors? “A total of 4 SNPs as instruments from Outdoors(winter).

The GWAS data for Computer was obtained from the UK Biobank (ukb-b-4522), which conducted a survey on Computer among 360,895 individuals. Classify the response to the question “In a typical DAY, how many hours do you spend using the computer? (Do not include using a computer at work; put 0 if you do not spend any time doing it)” A total of 78 SNPs as instruments from Computer.

The GWAS data for TV was obtained from the UK Biobank (ukb-b-5192), which conducted a survey on TV among 437,887 individuals. Classify the response to the question “In a typical DAY, how many hours do you spend watching TV? (Put 0 if you do not spend any time doing it)” A total of 103 SNPs as instruments from TV.

The GWAS data for AD was obtained from the IEU OpenGWAS project (ieu-b-5067), which conducted among 488,285 individuals, adjusted for age, sex, genotyping chip, and the first 10 genetic principal components. The details have been previously elucidated (Larsson et al., 2022).

### 2.3 Statistical analyses

**Figure 1:**
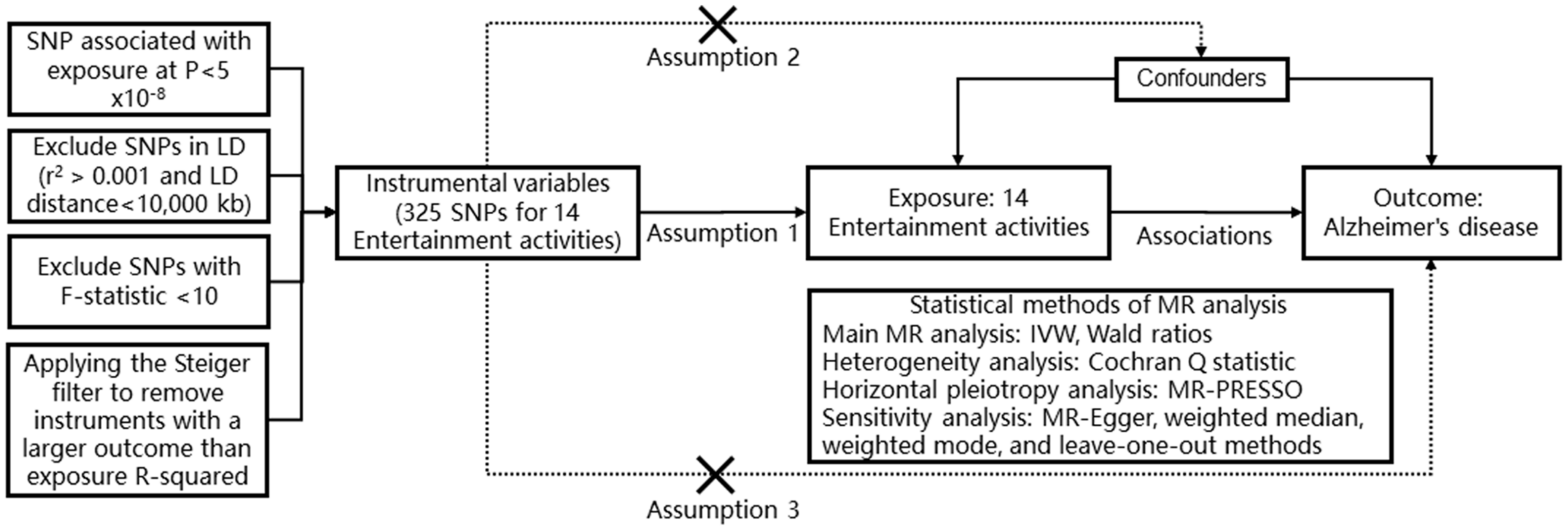
The design and flowchart of MR analysis in our study. This schematic representation highlights the research inquiry, analysis workflow, and utilized data in our study. Leveraging large-scale publicly available genome-wide association study (GWAS) summary statistics data, we conducted Mendelian randomization (MR) analysis to investigate the causal relationship between 14 entertainment activities and Alzheimer’s disease. Assumption 1: Sensitivity analysis was employed to validate the robustness of our MR findings. Assumption 2: The variant is significantly associated with the exposure. The variant is not associated with any confounder of the exposure-outcome association. Assumption 3: The variant does not affect the outcome, except possibly other biological pathways (i.e., horizontal pleiotropic effect) with the exposure SNP, single nucleotide polymorphism; MR-PRESSO, Mendelian randomization pleiotropy residual sum and outlier; MR, Mendelian randomization; IVW, inverse variance weighted.

In these serial two-sample MR analyses, we employed the inverse variance weighted (IVW) random effects method as a meta-analysis of variant-specific Wald ratios for each SNP to estimate the combined causal effect in both directions. The IVW method assumes independence and validity of IV (Smith and Ebrahim, 2003). However, it may overlook mediated effects or potential pleiotropy from other risk factors, leading to bias and violation of IV assumptions when horizontal pleiotropy exists among instrument SNPs (Bowden et al., 2015). Therefore, we additionally applied MR-Egger, weighted median, and weighted mode methods to assess robustness (Sanderson et al., 2022). The MR-Egger method assumes no association between the magnitude of pleiotropic effects and the strength of genetic-phenotype associations across all instruments (Bowden et al., 2015). Weighted mode requires a valid subset with consistent causal effects while weighted median assigns 50% weight to variables from valid instruments (Bowden et al., 2016). These supplementary analyses evaluate how alternative specifications affect MR results by considering possible pleiotropic effects. To detect heterogeneity, we used Cochran’s Q statistic with significance set at *p* < 0.05. Furthermore, we assessed horizontal pleiotropy using the MR-PRSSO analysis where *p* < 0.05 indicates its presence. All statistical analyses were performed using R 3.6.0 with the TwoSampleMR package (RRID: SCR_019010) (Figure 1).

## 3 Results

**Figure 2:**
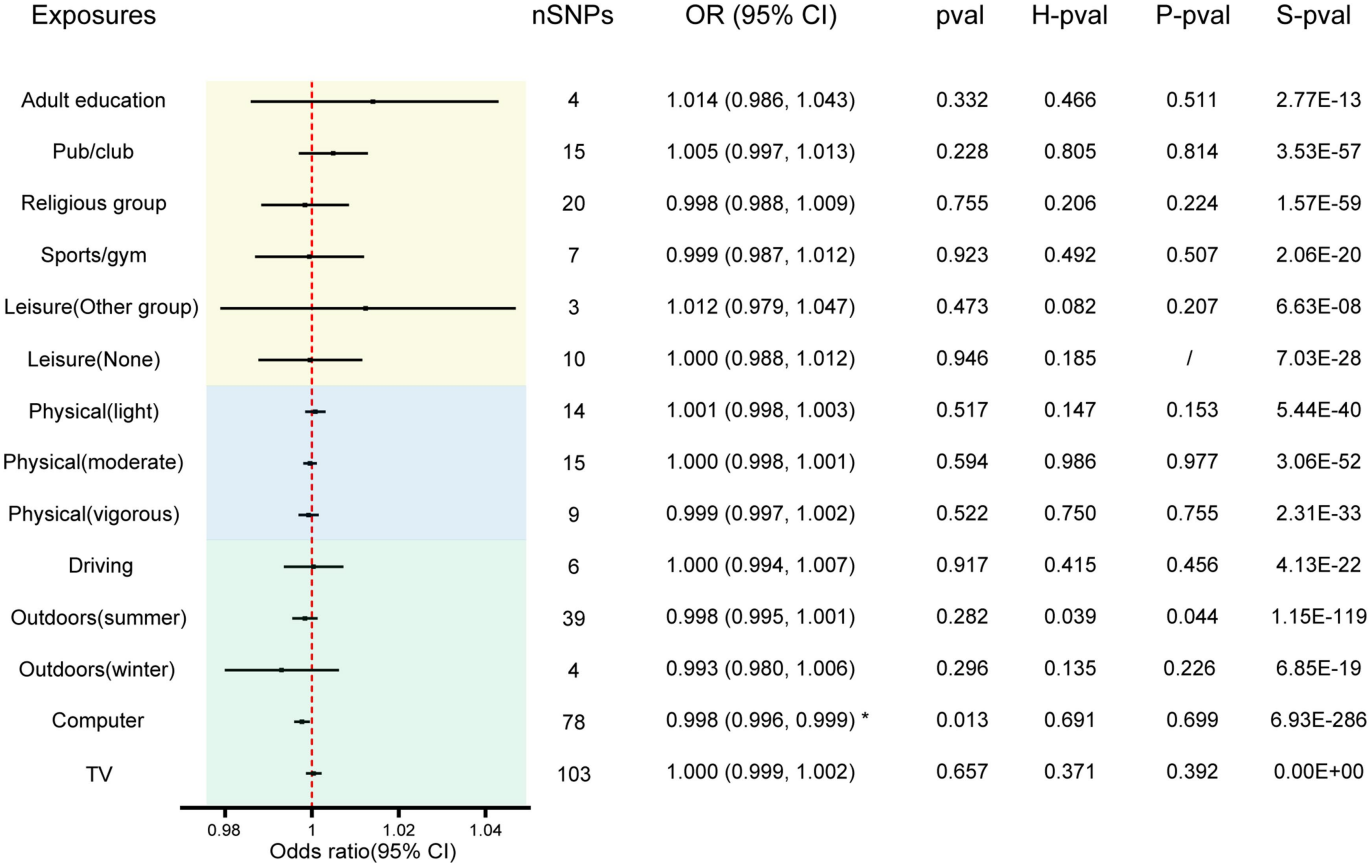
The effect of entertainment activities on AD. Results are from the IVW method of the two-sample MR analysis. * Indicates significant association with causal effect. The size of the box represents the weight of the study. SNP, single nucleotide polymorphism; OR, Odds ratios; CI, Confidence interval; H-pval, heterogeneity *p*-value; P-pval, horizontal pleiotropy *p*-value; S-pval, Steiger test *p*-value.

**Figure 3:**
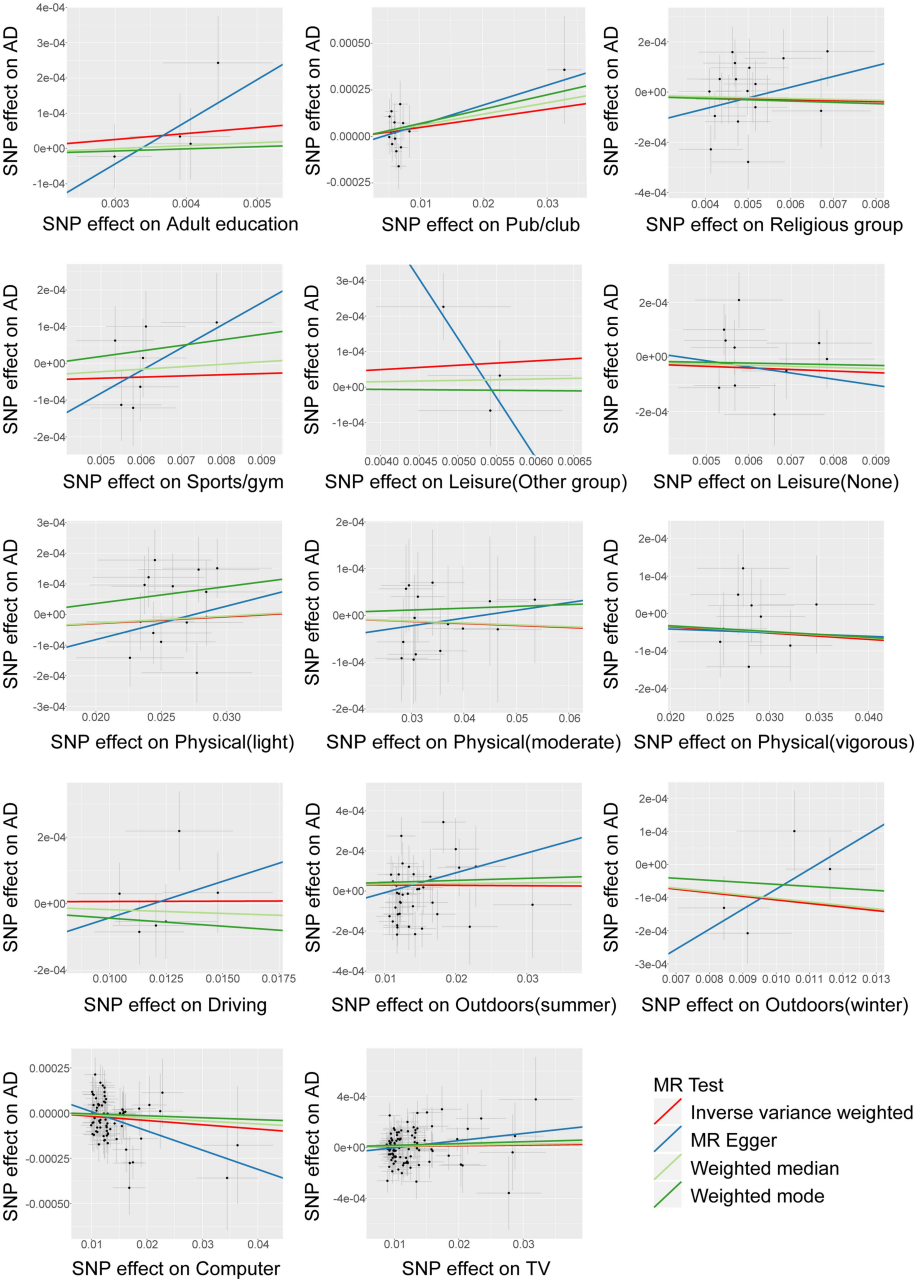
Assessment of significant causality in genetic associations between entertainment activities factors and AD. Each point indicates the associated change in trait level and the increased risk for each variant. The horizontal coordinate represents how much genetic variation is associated with exposure factors, and the vertical coordinate represents how much genetic variation is associated with disease risk. Horizontal and vertical lines through each point indicate the corresponding 95% CI for genetic association. The colored lines show the trait slope of the results obtained by applying various different MR methods.

The F-statistic for all instrumental variables exceeds 10, indicating the absence of weak instrument bias in our analysis (Supplementary Table S1). We analyzed 14 entertainment activities for their associations with AD by the IVW random effects method. The forest plot of the association between the risk of suffering from AD and each of the entertainment activity is shown in Figure 2. Figure 3 shows scatterplots of the associations of each genetic variant plotted against their association with the corresponding outcomes for each genetic variant. We found a statistically significant causal effect of time spent using computer on AD (OR 0.998; 95% CI 0.996–0.999; *p* = 0.013). Sensitivity analyses found no evidence of heterogeneity in Cochran’s Q statistic (*p* = 0.691) and there was no horizontal pleiotropy effect in the MR-PRESSO analysis (*p* = 0.699). There were no significant correlations between leisure/social activities, weekly exercise, time spent driving, time spent outdoors in summer, time spent outdoors in winter, or time spent watching television with AD. Full results are provided in Supplementary Table S2. The leave–one–out sensitivity further corroborated the aforementioned conclusion (Supplementary Table S3).

## 4 Discussion

In summary, our two-sample MR study revealed a significant association between genetically predicted time spent using computers and the risk of AD. Our findings suggest that increased computer use is associated with a reduced risk of developing AD. However, we did not find evidence supporting the hypothesis that leisure or social activities, weekly exercise, time spent driving, time outdoors in summer, time outdoors in winter, or time spent watching television are causally related to the risk of AD.

The association between computer usage and AD is discernible, with prior studies offering insights into this relationship. Evidence suggests that computer training for individuals with AD can positively impact AD progression and enhance quality of life (Klimova et al., 2018). Additionally, meaningful computer engagement may contribute to preventing or delaying dementia (Liapis and Harding, 2017). Using computers often involves a range of cognitive activities such as information searching and problem-solving, which require active thought and brain operation, promoting neuronal connections and cognitive function stimulation. Long-term engagement in these activities may help maintain brain activity and potentially reduce the risk of AD. Computers also serve as a platform for social interaction through email, social media, etc., which can combat loneliness and improve mental health, potentially aiding in AD prevention (Chen and Schulz, 2016). Furthermore, computers offer flexible interventions that can be used by patients with limited physical capabilities. Additionally, computer use may be associated with factors such as education level, economic status, and type of occupation, which themselves could influence AD risk (Nguyen et al., 2016; Korologou-Linden et al., 2022). Further confirmation of computer use as a modifiable risk factor for AD will assist individuals and primary healthcare units in developing actionable strategies for AD prevention and control, offering unique advantages. However, a study indicated that overstimulation from prolonged screen time during brain development can increase the risk of neurodegenerative diseases in adulthood (Manwell et al., 2022). Therefore, it is crucial to balance the duration and manner of computer use, avoiding excessive dependence and sedentary habits. Combined with proper rest and exercise, computers can be valuable tools in our daily lives.

Our research findings also present some discrepancies with previous conclusions. While many studies have demonstrated positive effects of physical exercise, social engagement, and outdoor activities on AD, our study did not find significant correlations (Karssemeijer et al., 2017; Ciofi et al., 2022; Huuha et al., 2022; Joshi et al., 2024). For instance, higher levels of physical activity have been linked to a reduced risk of AD and may enhance cerebral blood flow, modulate amyloid beta turnover, and exert neuroprotective effects on the brain (De la Rosa et al., 2020; Huuha et al., 2022). There is also evidence suggesting that leisure and social activities can enhance cognitive health in individuals with or at risk of AD (De la Rosa et al., 2020; Joshi et al., 2024). Additionally, participating in outdoor activities can promote feelings of well-being, provide enjoyable sensory experiences, and strengthen community connections for people with AD (Ciofi et al., 2022). The inconsistency in study results may stem from the complex nature of AD as a neurological disorder, influenced by various factors that may not have been adequately considered or controlled in our study, thus impacting the accurate assessment of their impact on AD.

The study exhibits both strengths and limitations. On the positive side, it covers a broad spectrum of entertainment activity factors and boasts a large sample size. However, potential drawbacks include the long preclinical phases of AD or changes caused by AD that may have occurred before diagnosis (Dubois et al., 2016). We were limited to summary-level data rather than individual-specific data, precluding the assessment of specific genetic variants at the individual level. Furthermore, our study population consisted solely of individuals of European ancestry, and genetic structure and disease prevalence differ across ethnicities, potentially limiting the generalizability of our findings to other ethnic groups (Lake et al., 2023). Additionally, some traits in our study had a relatively small number of genetic tools (< 10 SNPs each), posing a risk of weak instrument bias that could affect study precision and reliability. Looking ahead, with a deeper understanding of AD and rapid advancements in neuroscience, epidemiology, and related fields, future studies are likely to delve further into the correlation between entertainment activities and AD. Given that MR primarily assesses associated impacts, additional high-quality studies are imperative to investigate the potential benefits of entertainment activities on AD. This research promises to deepen our understanding of AD pathogenesis and provide robust scientific evidence to inform prevention and treatment strategies for the disease.

## 5 Conclusion

In conclusion, the results demonstrate a potential causal relationship between time spent using a computer and AD. This finding provides important insights for AD prevention and may facilitate the development of relevant prevention strategies. Nevertheless, further interventional trials are needed to elucidate the underlying mechanisms.

## Data availability statement

The original contributions presented in the study are included in the article/Supplementary material, further inquiries can be directed to the corresponding authors.

## Ethics statement

Ethical approval was not required for the study involving humans in accordance with the local legislation and institutional requirements. Written informed consent to participate in this study was not required from the participants or the participants’ legal guardians/next of kin in accordance with the national legislation and the institutional requirements.

## Author contributions

TL: Data curation, Funding acquisition, Writing – original draft, Writing – review & editing. LW: Data curation, Writing – original draft, Formal analysis. YZ: Data curation, Writing – original draft. HL: Methodology, Supervision, Writing – review & editing. JL: Data curation, Funding acquisition, Methodology, Writing – original draft, Writing – review & editing.
