# Supplementary material for: Entertainment activities and the risk of Alzheimer’s disease: a Mendelian randomization analysis
Source: Front Aging Neurosci. 2024 Jun 4;16:1419317. doi: 10.3389/fnagi.2024.1419317 (PMC11183303; doi:10.3389/fnagi.2024.1419317)
Supplement: Supplementary file 1 [file Table_1.docx]

**STROBE-MR checklist of recommended items to address in reports of Mendelian randomization studies**^1^ ^2^

| **Item No.** | **Section** | **Checklist item** | **Page No.** | **Relevant text from manuscript** |
| --- | --- | --- | --- | --- |
| 1 | **TITLE and ABSTRACT** | Indicate Mendelian randomization (MR) as the study’s design in the title and/or the abstract if that is a main purpose of the study | Article | Entertainment activities and the risk of Alzheimer's disease: a Mendelian Randomization Analysis |
|  | **INTRODUCTION** |  |  |  |
| 2 | **Background** | Explain the scientific background and rationale for the reported study. What is the exposure? Is a potential causal relationship between exposure and outcome plausible? Justify why MR is a helpful method to address the study question | Article | Mendelian randomization (MR) offers a methodological approach within epidemiology that uses genetic variants as instrumental variables (IV) to infer causality between risk factors and the outcome of interest. MR provides unbiased estimates of causality because genetic variants are inherited randomly from parents to offspring, independent of potential confounders. Therefore, this analytical method yields more definitive evidence regarding causality. |
| 3 | **Objectives** | State specific objectives clearly, including pre-specified causal hypotheses (if any). State that MR is a method that, under specific assumptions, intends to estimate causal effects | Article | The objective of this study is to investigate the influence of entertainment activities on AD within individual lifestyles. Here, we conducted a two-sample MR analysis to evaluate the causal impact of 14 entertainment activity factors on the risk of AD. The findings from this study could offer significant insights for preventing AD and contribute to the development of relevant prevention strategies. |
|  | **METHODS** |  |  |  |
| 4 | **Study design and data sources** | Present key elements of the study design early in the article. Consider including a table listing sources of data for all phases of the study. For each data source contributing to the analysis, describe the following: |  |  |
|  | a) | Setting: Describe the study design and the underlying population, if possible. Describe the setting, locations, and relevant dates, including periods of recruitment, exposure, follow-up, and data collection, when available. | Article | 2.1 Study design and data sources |
|  | b) | Participants: Give the eligibility criteria, and the sources and methods of selection of participants. Report the sample size, and whether any power or sample size calculations were carried out prior to the main analysis | Article | Table 1 |
|  | c) | Describe measurement, quality control and selection of genetic variants | Article | 2.2 Instrumental variable selection |
|  | d) | For each exposure, outcome, and other relevant variables, describe methods of assessment and diagnostic criteria for diseases | Article | 2.2 Instrumental variable selection |
|  | e) | Provide details of ethics committee approval and participant informed consent, if relevant | / | / |
| 5 | **Assumptions** | Explicitly state the three core IV assumptions for the main analysis (relevance, independence and exclusion restriction) as well assumptions for any additional or sensitivity analysis | Article | 2.3 Statistical analyses |
| 6 | **Statistical methods: main analysis** | Describe statistical methods and statistics used |  |  |
|  | a) | Describe how quantitative variables were handled in the analyses (i.e., scale, units, model) | Article | 2.2 Instrumental variable selection |
|  | b) | Describe how genetic variants were handled in the analyses and, if applicable, how their weights were selected | Article | 2.2 Instrumental variable selection |
|  | c) | Describe the MR estimator (e.g. two-stage least squares, Wald ratio) and related statistics. Detail the included covariates and, in case of two-sample MR, whether the same covariate set was used for adjustment in the two samples | Article | 2.3 Statistical analyses |
|  | d) | Explain how missing data were addressed | / | / |
|  | e) | If applicable, indicate how multiple testing was addressed | / | / |
| 7 | **Assessment of assumptions** | Describe any methods or prior knowledge used to assess the assumptions or justify their validity | Article | 2.3 Statistical analyses |
| 8 | **Sensitivity analyses and additional analyses** | Describe any sensitivity analyses or additional analyses performed (e.g. comparison of effect estimates from different approaches, independent replication, bias analytic techniques, validation of instruments, simulations) | Article | 2.3 Statistical analyses |
| 9 | **Software and pre-registration** |  |  |  |
|  | a) | Name statistical software and package(s), including version and settings used | Article | 2.3 Statistical analyses |
|  | b) | State whether the study protocol and details were pre-registered (as well as when and where) | Article | 2.3 Statistical analyses |
|  | **RESULTS** |  |  |  |
| 10 | **Descriptive data** |  |  |  |
|  | a) | Report the numbers of individuals at each stage of included studies and reasons for exclusion. Consider use of a flow diagram | Article | Fig 1 |
|  | b) | Report summary statistics for phenotypic exposure(s), outcome(s), and other relevant variables (e.g. means, SDs, proportions) | Article | Table 1 |
|  | c) | If the data sources include meta-analyses of previous studies, provide the assessments of heterogeneity across these studies | / | / |
|  | d) | For two-sample MR:  i.  Provide justification of the similarity of the genetic variant-exposure associations between the exposure and outcome samples  ii.  Provide information on the number of individuals who overlap between the exposure and outcome studies | Article | 2.2 Instrumental variable selection |
| 11 | **Main results** |  |  |  |
|  | a) | Report the associations between genetic variant and exposure, and between genetic variant and outcome, preferably on an interpretable scale | Supplementary | Supplementary Table S2 |
|  | b) | Report MR estimates of the relationship between exposure and outcome, and the measures of uncertainty from the MR analysis, on an interpretable scale, such as odds ratio or relative risk per SD difference | Supplementary | Supplementary Table S2 |
|  | c) | If relevant, consider translating estimates of relative risk into absolute risk for a meaningful time period | / | / |
|  | d) | Consider plots to visualize results (e.g. forest plot, scatterplot of associations between genetic variants and outcome versus between genetic variants and exposure) | Article | Fig 2-Fig 3 |
| 12 | **Assessment of assumptions** |  |  |  |
|  | a) | Report the assessment of the validity of the assumptions | / | / |
|  | b) | Report any additional statistics (e.g., assessments of heterogeneity across genetic variants, such as *I^2^*, Q statistic or E-value) | Supplementary | Supplementary Table S2 |
| 13 | **Sensitivity analyses and additional analyses** |  |  |  |
|  | a) | Report any sensitivity analyses to assess the robustness of the main results to violations of the assumptions | / | Figure 2 |
|  | b) | Report results from other sensitivity analyses or additional analyses | / | Figure 2 |
|  | c) | Report any assessment of direction of causal relationship (e.g., bidirectional MR) | / | Figure 2 |
|  | d) | When relevant, report and compare with estimates from non-MR analyses | / | / |
|  | e) | Consider additional plots to visualize results (e.g., leave-one-out analyses) | Supplementary | Supplementary Table S3 |
|  | **DISCUSSION** |  |  |  |
| 14 | **Key results** | Summarize key results with reference to study objectives | Article | In summary, our two-sample MR study revealed a significant association between genetically predicted time spent using computers and the risk of AD. Our findings suggest that increased computer use is associated with a reduced risk of developing AD. However, we did not find evidence supporting the hypothesis that leisure or social activities, weekly exercise, time spent driving, time outdoors in summer, time outdoors in winter, or time spent watching television are causally related to the risk of AD. |
| 15 | **Limitations** | Discuss limitations of the study, taking into account the validity of the IV assumptions, other sources of potential bias, and imprecision. Discuss both direction and magnitude of any potential bias and any efforts to address them | Article | However, potential drawbacks include the long preclinical phases of AD or changes caused by AD that may have occurred before diagnosis(30). We were limited to summary-level data rather than individual-specific data, precluding the assessment of specific genetic variants at the individual level. Furthermore, our study population consisted solely of individuals of European ancestry, and genetic structure and disease prevalence differ across ethnicities, potentially limiting the generalizability of our findings to other ethnic groups(31). Additionally, some traits in our study had a relatively small number of genetic tools (< 10 SNPs each), posing a risk of weak instrument bias that could affect study precision and reliability. |
| 16 | **Interpretation** |  |  |  |
|  | a) | Meaning: Give a cautious overall interpretation of results in the context of their limitations and in comparison with other studies | Article | Looking ahead, with a deeper understanding of AD and rapid advancements in neuroscience, epidemiology, and related fields, future studies are likely to delve further into the correlation between entertainment activities and AD. Given that MR primarily assesses associated impacts, additional high-quality studies are imperative to investigate the potential benefits of entertainment activities on AD. This research promises to deepen our understanding of AD pathogenesis and provide robust scientific evidence to inform prevention and treatment strategies for the disease. |
|  | b) | Mechanism: Discuss underlying biological mechanisms that could drive a potential causal relationship between the investigated exposure and the outcome, and whether the gene-environment equivalence assumption is reasonable. Use causal language carefully, clarifying that IV estimates may provide causal effects only under certain assumptions | Article | Evidence suggests that computer training for individuals with AD can positively impact AD progression and enhance quality of life(20). Additionally, meaningful computer engagement may contribute to preventing or delaying dementia(21). Using computers often involves a range of cognitive activities such as information searching and problem-solving, which require active thought and brain operation, promoting neuronal connections and cognitive function stimulation. Long-term engagement in these activities may help maintain brain activity and potentially reduce the risk of AD. Computers also serve as a platform for social interaction through email, social media, etc., which can combat loneliness and improve mental health, potentially aiding in AD prevention(22). Furthermore, computers offer flexible interventions that can be used by patients with limited physical capabilities. Additionally, computer use may be associated with factors such as education level, economic status, and type of occupation, which themselves could influence AD risk(23, 24). |
|  | c) | Clinical relevance: Discuss whether the results have clinical or public policy relevance, and to what extent they inform effect sizes of possible interventions | Article | Further confirmation of computer use as a modifiable risk factor for AD will assist individuals and primary healthcare units in developing actionable strategies for AD prevention and control, offering unique advantages. |
| 17 | **Generalizability** | Discuss the generalizability of the study results (a) to other populations, (b) across other exposure periods/timings, and (c) across other levels of exposure | Article | Looking ahead, with a deeper understanding of AD and rapid advancements in neuroscience, epidemiology, and related fields, future studies are likely to delve further into the correlation between entertainment activities and AD. Given that MR primarily assesses associated impacts, additional high-quality studies are imperative to investigate the potential benefits of entertainment activities on AD. This research promises to deepen our understanding of AD pathogenesis and provide robust scientific evidence to inform prevention and treatment strategies for the disease. |
|  | **OTHER INFORMATION** |  |  |  |
| 18 | **Funding** | Describe sources of funding and the role of funders in the present study and, if applicable, sources of funding for the databases and original study or studies on which the present study is based | Article | This work was supported by the National Natural Science Foundation of China (82303723), Sichuan Science and Technology Program (2023NSFSC1886), the Third People’s Hospital of Chengdu Scientific Research Project (CSY-YN-01-2023-031, CSY-YN-01-2023-045). |
| 19 | **Data and data sharing** | Provide the data used to perform all analyses or report where and how the data can be accessed, and reference these sources in the article. Provide the statistical code needed to reproduce the results in the article, or report whether the code is publicly accessible and if so, where | Article | 2.1 Study design and data sources |
| 20 | **Conflicts of Interest** | All authors should declare all potential conflicts of interest | Article | The authors declare that the research was conducted in the absence of any commercial or financial relationships that could be construed as a potential conflict of interest. |

This checklist is copyrighted by the Equator Network under the Creative Commons Attribution 3.0 Unported (CC BY 3.0) license.

1. Skrivankova VW, Richmond RC, Woolf BAR, Yarmolinsky J, Davies NM, Swanson SA, et al. Strengthening the Reporting of Observational Studies in Epidemiology using Mendelian Randomization (STROBE-MR) Statement. JAMA. 2021;under review.

2. Skrivankova VW, Richmond RC, Woolf BAR, Davies NM, Swanson SA, VanderWeele TJ, et al. Strengthening the Reporting of Observational Studies in Epidemiology using Mendelian Randomisation (STROBE-MR): Explanation and Elaboration. BMJ. 2021;375:n2233.
