# Supplementary material for: Entertainment activities and the risk of Alzheimer’s disease: a Mendelian randomization analysis
Source: Front Aging Neurosci. 2024 Jun 4;16:1419317. doi: 10.3389/fnagi.2024.1419317 (PMC11183303; doi:10.3389/fnagi.2024.1419317)
Supplement: Supplementary file 2 [file Data_Sheet_1.DOCX]

Supplementary Material

# Supplementary Table

**Supplementary Table S1 Detailed information of instrumental variables for entertainment activities on Alzheimer's disease in MR analysis.**

| SNP | Effect  allele | Other  allele | chr | pos | Sample  size | ID | R^2^ | F-statistics |
| --- | --- | --- | --- | --- | --- | --- | --- | --- |
| rs1491872 | T | C | 11 | 27792891 | 440512 | ukb-b-151 | 0.000292 | 33.72454 |
| rs2005617 | C | T | 9 | 33791164 | 440512 | ukb-b-151 | 0.000294 | 34.38041 |
| rs2189464 | T | C | 7 | 8633758 | 440512 | ukb-b-151 | 0.000269 | 31.03095 |
| rs2764261 | G | A | 6 | 108927842 | 440512 | ukb-b-151 | 0.000365 | 42.55239 |
| rs328900 | T | C | 7 | 35020280 | 440512 | ukb-b-151 | 0.000366 | 42.68695 |
| rs382210 | A | G | 3 | 84966018 | 440512 | ukb-b-151 | 0.000359 | 41.93863 |
| rs6955240 | A | G | 7 | 133581873 | 440512 | ukb-b-151 | 0.000491 | 57.17936 |
| rs7072776 | G | A | 10 | 22032942 | 440512 | ukb-b-151 | 0.0003 | 34.85411 |
| rs7749823 | C | A | 6 | 26158079 | 440512 | ukb-b-151 | 0.000306 | 35.82492 |
| rs1125000 | T | C | 6 | 26287256 | 461369 | ukb-b-1553 | 4.47E-06 | 31.63657 |
| rs6997 | T | C | 3 | 49453834 | 461369 | ukb-b-1553 | 7.01E-06 | 48.72785 |
| rs72951028 | T | C | 18 | 30832603 | 461369 | ukb-b-1553 | 4.79E-06 | 33.29254 |
| rs9930477 | G | C | 16 | 9144595 | 461369 | ukb-b-1553 | 4.49E-06 | 30.45423 |
| rs11057408 | T | G | 12 | 124464836 | 310555 | ukb-b-3793 | 5.69E-05 | 32.52962 |
| rs12069474 | C | A | 1 | 98438135 | 310555 | ukb-b-3793 | 5.35E-05 | 30.38093 |
| rs12921753 | T | C | 16 | 30018720 | 310555 | ukb-b-3793 | 6.96E-05 | 39.76135 |
| rs2090660 | T | C | 2 | 136818719 | 310555 | ukb-b-3793 | 5.35E-05 | 30.31127 |
| rs2588917 | A | C | 10 | 63524979 | 310555 | ukb-b-3793 | 5.39E-05 | 30.47706 |
| rs975303 | G | A | 6 | 19028788 | 310555 | ukb-b-3793 | 6.48E-05 | 36.75743 |
| rs11712056 | C | T | 3 | 49914397 | 461369 | ukb-b-4000 | 1.76E-05 | 38.73281 |
| rs13002862 | A | C | 2 | 137613935 | 461369 | ukb-b-4000 | 1.38E-05 | 30.45788 |
| rs17315037 | C | T | 1 | 62803731 | 461369 | ukb-b-4000 | 1.47E-05 | 32.02732 |
| rs2499760 | A | C | 6 | 33945972 | 461369 | ukb-b-4000 | 1.43E-05 | 31.03763 |
| rs62263912 | A | G | 3 | 85648246 | 461369 | ukb-b-4000 | 1.75E-05 | 38.02457 |
| rs6478444 | G | A | 9 | 122681580 | 461369 | ukb-b-4000 | 1.38E-05 | 30.07081 |
| rs7228990 | G | C | 18 | 75887984 | 461369 | ukb-b-4000 | 1.37E-05 | 29.92059 |
| rs11917431 | T | C | 3 | 49644012 | 461369 | ukb-b-4077 | 1.29E-05 | 35.20937 |
| rs17824247 | C | T | 2 | 144152539 | 461369 | ukb-b-4077 | 1.12E-05 | 30.40782 |
| rs2773485 | C | T | 10 | 11123697 | 461369 | ukb-b-4077 | 1.25E-05 | 33.78951 |
| rs10978543 | G | A | 9 | 109328165 | 461369 | ukb-b-4171 | 1.42E-05 | 34.6288 |
| rs11066099 | G | C | 12 | 112392036 | 461369 | ukb-b-4171 | 1.22E-05 | 29.88405 |
| rs12103006 | G | A | 16 | 24726237 | 461369 | ukb-b-4171 | 1.24E-05 | 30.07281 |
| rs1229984 | C | T | 4 | 100239319 | 461369 | ukb-b-4171 | 5.67E-05 | 141.4657 |
| rs12472555 | G | T | 2 | 162816728 | 461369 | ukb-b-4171 | 1.50E-05 | 36.29342 |
| rs139920 | T | C | 22 | 40726749 | 461369 | ukb-b-4171 | 1.89E-05 | 45.91263 |
| rs2661863 | C | T | 1 | 8452725 | 461369 | ukb-b-4171 | 1.29E-05 | 31.54802 |
| rs35864506 | G | T | 4 | 100301700 | 461369 | ukb-b-4171 | 1.51E-05 | 35.93039 |
| rs36030660 | C | T | 18 | 57735945 | 461369 | ukb-b-4171 | 1.38E-05 | 33.58899 |
| rs56207132 | T | C | 1 | 51040826 | 461369 | ukb-b-4171 | 1.26E-05 | 30.34802 |
| rs61873510 | T | G | 10 | 102626510 | 461369 | ukb-b-4171 | 1.51E-05 | 35.07743 |
| rs6969458 | A | G | 7 | 153489725 | 461369 | ukb-b-4171 | 2.59E-05 | 61.93801 |
| rs754204 | T | C | 13 | 110411568 | 461369 | ukb-b-4171 | 1.29E-05 | 30.71892 |
| rs7545161 | T | C | 1 | 97474454 | 461369 | ukb-b-4171 | 1.42E-05 | 34.54638 |
| rs9401593 | C | A | 6 | 98549801 | 461369 | ukb-b-4171 | 1.41E-05 | 34.07564 |
| rs10208088 | T | C | 2 | 221055873 | 360895 | ukb-b-4522 | 5.11E-05 | 30.68537 |
| rs1037091 | T | C | 2 | 155652357 | 360895 | ukb-b-4522 | 0.000105 | 60.91812 |
| rs10518019 | G | A | 4 | 67959875 | 360895 | ukb-b-4522 | 4.93E-05 | 29.73799 |
| rs11259902 | A | C | 15 | 83886529 | 360895 | ukb-b-4522 | 5.34E-05 | 31.89512 |
| rs112600282 | G | A | 2 | 156895797 | 360895 | ukb-b-4522 | 5.62E-05 | 33.48136 |
| rs113851275 | A | G | 9 | 98297220 | 360895 | ukb-b-4522 | 6.66E-05 | 39.99395 |
| rs11634155 | C | T | 15 | 26693096 | 360895 | ukb-b-4522 | 6.01E-05 | 35.55579 |
| rs11652437 | A | C | 17 | 79338469 | 360895 | ukb-b-4522 | 7.15E-05 | 41.77254 |
| rs117405403 | C | G | 1 | 197744098 | 360895 | ukb-b-4522 | 6.56E-05 | 39.55704 |
| rs11749912 | G | A | 5 | 88065628 | 360895 | ukb-b-4522 | 8.11E-05 | 48.25616 |
| rs11766392 | T | G | 7 | 69838127 | 360895 | ukb-b-4522 | 6.61E-05 | 39.58462 |
| rs11942953 | C | T | 4 | 163753973 | 360895 | ukb-b-4522 | 5.44E-05 | 32.32401 |
| rs12128707 | G | A | 1 | 72588119 | 360895 | ukb-b-4522 | 5.63E-05 | 33.51754 |
| rs12145677 | A | G | 1 | 110023610 | 360895 | ukb-b-4522 | 0.000101 | 60.76214 |
| rs1229984 | C | T | 4 | 100239319 | 360895 | ukb-b-4522 | 6.32E-05 | 38.9107 |
| rs12521638 | G | A | 5 | 166458770 | 360895 | ukb-b-4522 | 5.02E-05 | 30.05579 |
| rs12553324 | G | C | 9 | 23347865 | 360895 | ukb-b-4522 | 0.000148 | 89.02638 |
| rs12706626 | A | G | 7 | 124531370 | 360895 | ukb-b-4522 | 5.12E-05 | 30.7618 |
| rs12820967 | C | T | 12 | 38921745 | 360895 | ukb-b-4522 | 6.18E-05 | 36.55661 |
| rs12946454 | T | A | 17 | 43208121 | 360895 | ukb-b-4522 | 5.88E-05 | 35.12351 |
| rs13262595 | G | A | 8 | 143316970 | 360895 | ukb-b-4522 | 0.00012 | 72.37065 |
| rs13422733 | T | C | 2 | 102010245 | 360895 | ukb-b-4522 | 5.04E-05 | 30.11499 |
| rs136553 | T | C | 22 | 27255675 | 360895 | ukb-b-4522 | 6.01E-05 | 35.99414 |
| rs1395020 | A | G | 4 | 139690326 | 360895 | ukb-b-4522 | 5.11E-05 | 30.46566 |
| rs1448355 | T | C | 11 | 131286685 | 360895 | ukb-b-4522 | 7.09E-05 | 42.26268 |
| rs1469249 | A | G | 5 | 113837198 | 360895 | ukb-b-4522 | 5.60E-05 | 33.2767 |
| rs147543875 | T | C | 10 | 101624164 | 360895 | ukb-b-4522 | 5.85E-05 | 31.99315 |
| rs1648906 | A | G | 18 | 35311651 | 360895 | ukb-b-4522 | 5.32E-05 | 31.4283 |
| rs166835 | T | C | 15 | 47716037 | 360895 | ukb-b-4522 | 5.78E-05 | 34.48102 |
| rs16912540 | G | A | 11 | 13271422 | 360895 | ukb-b-4522 | 5.91E-05 | 35.36278 |
| rs17167210 | A | G | 7 | 133339343 | 360895 | ukb-b-4522 | 6.39E-05 | 38.30653 |
| rs17789218 | C | T | 6 | 100600097 | 360895 | ukb-b-4522 | 5.97E-05 | 35.87171 |
| rs17862355 | G | T | 7 | 126970135 | 360895 | ukb-b-4522 | 6.20E-05 | 37.24284 |
| rs1987942 | C | T | 13 | 54004785 | 360895 | ukb-b-4522 | 5.78E-05 | 34.04666 |
| rs2032780 | C | T | 2 | 215073935 | 360895 | ukb-b-4522 | 7.36E-05 | 43.37398 |
| rs2068625 | C | T | 4 | 159856739 | 360895 | ukb-b-4522 | 8.46E-05 | 50.77582 |
| rs206965 | C | T | 12 | 120856332 | 360895 | ukb-b-4522 | 5.54E-05 | 33.29667 |
| rs2120461 | T | C | 1 | 8447722 | 360895 | ukb-b-4522 | 6.65E-05 | 40.00261 |
| rs2220599 | G | C | 5 | 7378854 | 360895 | ukb-b-4522 | 7.06E-05 | 42.1403 |
| rs246723 | G | A | 5 | 140519166 | 360895 | ukb-b-4522 | 5.50E-05 | 32.50236 |
| rs2588543 | T | C | 4 | 37000406 | 360895 | ukb-b-4522 | 5.25E-05 | 31.28674 |
| rs2734833 | A | G | 11 | 113292920 | 360895 | ukb-b-4522 | 7.31E-05 | 43.72567 |
| rs2748985 | C | T | 1 | 1853184 | 360895 | ukb-b-4522 | 7.75E-05 | 46.44722 |
| rs2756121 | A | T | 14 | 103989581 | 360895 | ukb-b-4522 | 6.21E-05 | 37.00188 |
| rs2761438 | G | A | 1 | 110752139 | 360895 | ukb-b-4522 | 6.03E-05 | 36.27281 |
| rs28710456 | C | T | 4 | 152667171 | 360895 | ukb-b-4522 | 5.41E-05 | 32.42607 |
| rs306755 | C | T | 20 | 3099752 | 360895 | ukb-b-4522 | 5.37E-05 | 32.17852 |
| rs34238696 | G | A | 5 | 161356241 | 360895 | ukb-b-4522 | 5.39E-05 | 32.26836 |
| rs3730399 | G | A | 16 | 67229019 | 360895 | ukb-b-4522 | 6.26E-05 | 37.50732 |
| rs4704043 | T | C | 5 | 72159179 | 360895 | ukb-b-4522 | 5.51E-05 | 33.07519 |
| rs4852252 | C | T | 2 | 71539301 | 360895 | ukb-b-4522 | 5.11E-05 | 30.71843 |
| rs56229818 | C | T | 15 | 58662232 | 360895 | ukb-b-4522 | 5.12E-05 | 30.71542 |
| rs6028090 | A | G | 20 | 59856465 | 360895 | ukb-b-4522 | 7.76E-05 | 45.87003 |
| rs613872 | T | G | 18 | 53210302 | 360895 | ukb-b-4522 | 7.07E-05 | 42.32958 |
| rs6449708 | C | T | 5 | 50851575 | 360895 | ukb-b-4522 | 5.57E-05 | 33.30223 |
| rs6780848 | G | T | 3 | 8179920 | 360895 | ukb-b-4522 | 5.09E-05 | 30.54167 |
| rs6935828 | T | C | 6 | 140811367 | 360895 | ukb-b-4522 | 5.10E-05 | 30.64998 |
| rs7020477 | G | A | 9 | 116827760 | 360895 | ukb-b-4522 | 5.61E-05 | 33.54797 |
| rs707926 | A | G | 6 | 31748820 | 360895 | ukb-b-4522 | 5.40E-05 | 32.54366 |
| rs7209653 | C | T | 17 | 19882084 | 360895 | ukb-b-4522 | 7.46E-05 | 44.77185 |
| rs7281293 | C | A | 21 | 34291496 | 360895 | ukb-b-4522 | 5.94E-05 | 35.18931 |
| rs72828532 | C | T | 6 | 19065342 | 360895 | ukb-b-4522 | 7.24E-05 | 43.36738 |
| rs72847500 | C | T | 6 | 37643909 | 360895 | ukb-b-4522 | 5.31E-05 | 31.62324 |
| rs7288455 | G | A | 22 | 39966547 | 360895 | ukb-b-4522 | 5.46E-05 | 32.64687 |
| rs73578186 | T | C | 9 | 126334485 | 360895 | ukb-b-4522 | 6.62E-05 | 39.26453 |
| rs7526112 | G | T | 1 | 93747683 | 360895 | ukb-b-4522 | 5.43E-05 | 32.65323 |
| rs75550998 | T | G | 2 | 146486095 | 360895 | ukb-b-4522 | 5.08E-05 | 29.89314 |
| rs7564844 | A | G | 2 | 215335556 | 360895 | ukb-b-4522 | 6.39E-05 | 38.45278 |
| rs76112266 | G | C | 2 | 201087157 | 360895 | ukb-b-4522 | 7.32E-05 | 43.86229 |
| rs7630869 | T | C | 3 | 49522543 | 360895 | ukb-b-4522 | 0.00011 | 66.2491 |
| rs76824303 | C | A | 3 | 62459819 | 360895 | ukb-b-4522 | 7.52E-05 | 42.89134 |
| rs7904398 | T | C | 10 | 67954193 | 360895 | ukb-b-4522 | 5.20E-05 | 31.13555 |
| rs7968738 | A | G | 12 | 90281747 | 360895 | ukb-b-4522 | 5.99E-05 | 35.65886 |
| rs79720045 | C | T | 4 | 39797668 | 360895 | ukb-b-4522 | 7.82E-05 | 45.23061 |
| rs806795 | A | G | 6 | 26205293 | 360895 | ukb-b-4522 | 5.34E-05 | 32.23027 |
| rs8102851 | C | T | 19 | 32208909 | 360895 | ukb-b-4522 | 5.72E-05 | 33.38297 |
| rs9375188 | T | C | 6 | 98555272 | 360895 | ukb-b-4522 | 0.000126 | 74.89627 |
| rs9537571 | A | G | 13 | 57604700 | 360895 | ukb-b-4522 | 6.21E-05 | 37.22007 |
| rs11877152 | C | T | 18 | 42754468 | 461369 | ukb-b-4667 | 9.04E-06 | 33.87902 |
| rs12119422 | A | G | 1 | 242244878 | 461369 | ukb-b-4667 | 8.23E-06 | 30.53408 |
| rs12156017 | G | C | 8 | 9406837 | 461369 | ukb-b-4667 | 1.03E-05 | 38.40584 |
| rs12992090 | T | C | 2 | 100827340 | 461369 | ukb-b-4667 | 1.19E-05 | 44.72509 |
| rs1471093 | A | G | 3 | 108031094 | 461369 | ukb-b-4667 | 1.41E-05 | 52.07738 |
| rs17527878 | T | C | 18 | 53404986 | 461369 | ukb-b-4667 | 9.49E-06 | 35.36431 |
| rs1991083 | T | C | 2 | 23887437 | 461369 | ukb-b-4667 | 9.34E-06 | 34.6219 |
| rs3197999 | A | G | 3 | 49721532 | 461369 | ukb-b-4667 | 9.09E-06 | 33.97343 |
| rs332828 | A | G | 1 | 61742693 | 461369 | ukb-b-4667 | 1.33E-05 | 49.6833 |
| rs34402524 | G | T | 4 | 106196829 | 461369 | ukb-b-4667 | 1.03E-05 | 39.0919 |
| rs36104984 | A | G | 3 | 35691748 | 461369 | ukb-b-4667 | 8.67E-06 | 32.2841 |
| rs410671 | G | A | 5 | 87993198 | 461369 | ukb-b-4667 | 1.30E-05 | 47.8349 |
| rs6545977 | A | G | 2 | 63301164 | 461369 | ukb-b-4667 | 8.37E-06 | 31.31725 |
| rs6722794 | C | T | 2 | 124278619 | 461369 | ukb-b-4667 | 8.66E-06 | 32.18965 |
| rs6862251 | T | C | 5 | 167558838 | 461369 | ukb-b-4667 | 1.10E-05 | 41.03211 |
| rs6944796 | T | C | 7 | 104505787 | 461369 | ukb-b-4667 | 1.12E-05 | 41.48236 |
| rs699534 | C | A | 1 | 90942808 | 461369 | ukb-b-4667 | 1.08E-05 | 39.8796 |
| rs7388625 | T | A | 8 | 93218536 | 461369 | ukb-b-4667 | 8.16E-06 | 30.4756 |
| rs8020432 | A | C | 14 | 34025195 | 461369 | ukb-b-4667 | 8.08E-06 | 29.80658 |
| rs990702 | T | C | 7 | 97289021 | 461369 | ukb-b-4667 | 9.11E-06 | 33.61413 |
| rs10098073 | A | C | 8 | 143309504 | 440266 | ukb-b-4710 | 0.000468 | 37.40298 |
| rs11749912 | G | A | 5 | 88065628 | 440266 | ukb-b-4710 | 0.000411 | 32.95325 |
| rs11913445 | A | C | 22 | 20142513 | 440266 | ukb-b-4710 | 0.000383 | 30.45859 |
| rs2246122 | C | T | 13 | 44826508 | 440266 | ukb-b-4710 | 0.000401 | 32.18008 |
| rs3094622 | G | A | 6 | 30327952 | 440266 | ukb-b-4710 | 0.00067 | 54.32864 |
| rs3129962 | A | G | 6 | 32379383 | 440266 | ukb-b-4710 | 0.000455 | 36.88392 |
| rs34775997 | A | G | 15 | 95304276 | 440266 | ukb-b-4710 | 0.00038 | 30.64667 |
| rs4129572 | C | T | 7 | 133636888 | 440266 | ukb-b-4710 | 0.000608 | 48.81701 |
| rs4540651 | A | G | 1 | 154128672 | 440266 | ukb-b-4710 | 0.000396 | 32.02046 |
| rs4886868 | G | T | 15 | 74353561 | 440266 | ukb-b-4710 | 0.00045 | 35.44566 |
| rs7229874 | A | C | 18 | 6106730 | 440266 | ukb-b-4710 | 0.000395 | 31.34223 |
| rs7565480 | G | A | 2 | 200868926 | 440266 | ukb-b-4710 | 0.000391 | 31.67777 |
| rs9533455 | T | C | 13 | 43892830 | 440266 | ukb-b-4710 | 0.000409 | 33.16567 |
| rs997467 | C | T | 2 | 199182704 | 440266 | ukb-b-4710 | 0.000478 | 37.41728 |
| rs11183184 | C | T | 12 | 46110108 | 454783 | ukb-b-4886 | 0.000273 | 32.47367 |
| rs12568280 | T | C | 1 | 154122974 | 454783 | ukb-b-4886 | 0.000297 | 36.18829 |
| rs2011071 | G | A | 5 | 88741077 | 454783 | ukb-b-4886 | 0.000269 | 32.27205 |
| rs2220599 | G | C | 5 | 7378854 | 454783 | ukb-b-4886 | 0.000399 | 48.42781 |
| rs34654885 | A | G | 4 | 5232752 | 454783 | ukb-b-4886 | 0.000327 | 40.11503 |
| rs4588066 | A | G | 18 | 40672964 | 454783 | ukb-b-4886 | 0.000258 | 31.46592 |
| rs4641022 | G | A | 8 | 92803746 | 454783 | ukb-b-4886 | 0.000257 | 31.49405 |
| rs62172117 | A | G | 2 | 144168667 | 454783 | ukb-b-4886 | 0.000353 | 43.03066 |
| rs62422661 | T | G | 6 | 98545611 | 454783 | ukb-b-4886 | 0.000386 | 47.20362 |
| rs62482241 | A | G | 7 | 100235508 | 454783 | ukb-b-4886 | 0.000295 | 35.9426 |
| rs72931435 | G | A | 6 | 72751948 | 454783 | ukb-b-4886 | 0.000248 | 30.38977 |
| rs747344 | G | A | 2 | 23651300 | 454783 | ukb-b-4886 | 0.000248 | 30.27856 |
| rs7576657 | C | A | 2 | 60779478 | 454783 | ukb-b-4886 | 0.000336 | 40.32214 |
| rs9400239 | C | T | 6 | 108977663 | 454783 | ukb-b-4886 | 0.000247 | 30.25345 |
| rs12889064 | G | T | 14 | 39421742 | 461369 | ukb-b-5076 | 1.43E-05 | 31.11592 |
| rs13011181 | C | T | 2 | 100732446 | 461369 | ukb-b-5076 | 2.24E-05 | 48.77477 |
| rs2043145 | G | A | 18 | 50851107 | 461369 | ukb-b-5076 | 1.57E-05 | 34.34018 |
| rs2675638 | A | G | 10 | 63576286 | 461369 | ukb-b-5076 | 1.43E-05 | 31.42053 |
| rs3197999 | A | G | 3 | 49721532 | 461369 | ukb-b-5076 | 1.94E-05 | 42.30018 |
| rs35291206 | G | C | 5 | 145453377 | 461369 | ukb-b-5076 | 1.43E-05 | 30.67307 |
| rs410671 | G | A | 5 | 87993198 | 461369 | ukb-b-5076 | 1.44E-05 | 31.06957 |
| rs4247450 | C | A | 3 | 107624530 | 461369 | ukb-b-5076 | 1.43E-05 | 31.18448 |
| rs4470910 | T | C | 7 | 2071723 | 461369 | ukb-b-5076 | 1.75E-05 | 38.20261 |
| rs847687 | G | T | 17 | 48229441 | 461369 | ukb-b-5076 | 1.40E-05 | 30.69956 |
| rs10109061 | G | A | 8 | 144239859 | 437887 | ukb-b-5192 | 3.97E-05 | 30.67228 |
| rs10189857 | G | A | 2 | 60713235 | 437887 | ukb-b-5192 | 0.000106 | 83.71824 |
| rs10269099 | T | G | 7 | 126371011 | 437887 | ukb-b-5192 | 3.99E-05 | 31.41191 |
| rs10739499 | G | C | 9 | 120514261 | 437887 | ukb-b-5192 | 3.81E-05 | 30.04955 |
| rs10765777 | C | A | 11 | 95656385 | 437887 | ukb-b-5192 | 6.09E-05 | 47.73019 |
| rs11191129 | T | C | 10 | 103606543 | 437887 | ukb-b-5192 | 4.73E-05 | 37.4663 |
| rs11222919 | G | T | 11 | 131969663 | 437887 | ukb-b-5192 | 4.31E-05 | 33.74983 |
| rs11245482 | C | T | 10 | 126733546 | 437887 | ukb-b-5192 | 4.92E-05 | 38.77208 |
| rs114600294 | C | G | 3 | 181419367 | 437887 | ukb-b-5192 | 3.86E-05 | 30.62531 |
| rs114755463 | A | G | 5 | 152503110 | 437887 | ukb-b-5192 | 4.91E-05 | 38.09656 |
| rs115608101 | T | C | 11 | 107106532 | 437887 | ukb-b-5192 | 4.16E-05 | 32.59875 |
| rs11662211 | T | C | 18 | 77618869 | 437887 | ukb-b-5192 | 4.46E-05 | 35.24255 |
| rs11680095 | T | C | 2 | 181825956 | 437887 | ukb-b-5192 | 4.08E-05 | 31.61916 |
| rs11696187 | T | C | 20 | 58891882 | 437887 | ukb-b-5192 | 4.95E-05 | 39.02933 |
| rs11700249 | G | T | 20 | 11910800 | 437887 | ukb-b-5192 | 4.30E-05 | 33.86023 |
| rs11714337 | A | G | 3 | 71582521 | 437887 | ukb-b-5192 | 4.82E-05 | 37.85503 |
| rs11877758 | G | T | 18 | 35138110 | 437887 | ukb-b-5192 | 5.27E-05 | 41.28487 |
| rs11911112 | C | A | 21 | 40528346 | 437887 | ukb-b-5192 | 5.36E-05 | 42.2476 |
| rs12045585 | A | G | 1 | 243673099 | 437887 | ukb-b-5192 | 5.17E-05 | 39.0684 |
| rs12214364 | G | T | 6 | 67556372 | 437887 | ukb-b-5192 | 3.97E-05 | 30.23402 |
| rs12553324 | G | C | 9 | 23347865 | 437887 | ukb-b-5192 | 8.94E-05 | 70.5236 |
| rs1291871 | C | T | 10 | 11086083 | 437887 | ukb-b-5192 | 4.19E-05 | 32.86168 |
| rs13014947 | A | G | 2 | 193742999 | 437887 | ukb-b-5192 | 5.50E-05 | 42.66295 |
| rs13107325 | T | C | 4 | 103188709 | 437887 | ukb-b-5192 | 5.74E-05 | 45.32657 |
| rs1324491 | A | G | 1 | 60350616 | 437887 | ukb-b-5192 | 4.10E-05 | 32.36974 |
| rs1727332 | T | C | 12 | 123718301 | 437887 | ukb-b-5192 | 6.42E-05 | 50.60123 |
| rs17789218 | C | T | 6 | 100600097 | 437887 | ukb-b-5192 | 4.40E-05 | 34.84526 |
| rs180396 | T | C | 13 | 60437497 | 437887 | ukb-b-5192 | 3.82E-05 | 30.00743 |
| rs1826510 | A | G | 5 | 24800012 | 437887 | ukb-b-5192 | 4.61E-05 | 31.62104 |
| rs184332798 | A | G | 18 | 53373610 | 437887 | ukb-b-5192 | 3.92E-05 | 30.81494 |
| rs1993092 | C | T | 6 | 98689604 | 437887 | ukb-b-5192 | 4.31E-05 | 34.0514 |
| rs2073869 | T | C | 9 | 135763816 | 437887 | ukb-b-5192 | 5.29E-05 | 41.61059 |
| rs2106164 | C | T | 7 | 92661753 | 437887 | ukb-b-5192 | 4.39E-05 | 34.51327 |
| rs2185490 | C | A | 14 | 69732119 | 437887 | ukb-b-5192 | 4.15E-05 | 32.4708 |
| rs2240857 | G | T | 7 | 8010634 | 437887 | ukb-b-5192 | 6.15E-05 | 47.89037 |
| rs2283 | G | A | 5 | 106773623 | 437887 | ukb-b-5192 | 3.85E-05 | 30.59187 |
| rs2332818 | C | T | 14 | 27112432 | 437887 | ukb-b-5192 | 3.84E-05 | 30.17223 |
| rs2352984 | C | T | 3 | 49948728 | 437887 | ukb-b-5192 | 0.000191 | 151.7031 |
| rs2479968 | G | A | 13 | 111969328 | 437887 | ukb-b-5192 | 4.11E-05 | 30.92238 |
| rs249960 | G | A | 5 | 96164771 | 437887 | ukb-b-5192 | 4.15E-05 | 32.63276 |
| rs262890 | G | A | 5 | 62930015 | 437887 | ukb-b-5192 | 7.35E-05 | 58.0422 |
| rs263771 | A | C | 2 | 185921692 | 437887 | ukb-b-5192 | 5.17E-05 | 40.60192 |
| rs2646351 | A | G | 4 | 55701312 | 437887 | ukb-b-5192 | 3.94E-05 | 31.10149 |
| rs2678662 | G | T | 2 | 104446759 | 437887 | ukb-b-5192 | 5.92E-05 | 46.62083 |
| rs2725371 | G | A | 8 | 30854033 | 437887 | ukb-b-5192 | 7.45E-05 | 58.47891 |
| rs2857693 | T | G | 6 | 31588384 | 437887 | ukb-b-5192 | 5.27E-05 | 41.90662 |
| rs2906604 | C | T | 2 | 107624244 | 437887 | ukb-b-5192 | 5.41E-05 | 42.99394 |
| rs3138499 | C | A | 9 | 92219921 | 437887 | ukb-b-5192 | 6.00E-05 | 46.65829 |
| rs34811474 | A | G | 4 | 25408838 | 437887 | ukb-b-5192 | 6.14E-05 | 48.59846 |
| rs35797019 | G | A | 3 | 93987306 | 437887 | ukb-b-5192 | 4.05E-05 | 32.03995 |
| rs362312 | C | T | 4 | 3237644 | 437887 | ukb-b-5192 | 4.43E-05 | 34.89259 |
| rs3754970 | C | T | 2 | 162091836 | 437887 | ukb-b-5192 | 4.71E-05 | 36.82831 |
| rs3810496 | C | T | 20 | 62406886 | 437887 | ukb-b-5192 | 4.15E-05 | 32.48264 |
| rs4076457 | T | C | 15 | 78007213 | 437887 | ukb-b-5192 | 3.85E-05 | 30.29861 |
| rs4110177 | A | G | 5 | 88793281 | 437887 | ukb-b-5192 | 4.13E-05 | 32.37491 |
| rs4303732 | C | T | 2 | 100830040 | 437887 | ukb-b-5192 | 5.53E-05 | 43.77946 |
| rs4339469 | G | T | 6 | 98369230 | 437887 | ukb-b-5192 | 7.32E-05 | 57.86078 |
| rs4469687 | G | A | 1 | 184679019 | 437887 | ukb-b-5192 | 3.87E-05 | 30.6209 |
| rs4567133 | A | C | 9 | 22606560 | 437887 | ukb-b-5192 | 5.21E-05 | 40.82154 |
| rs4747438 | T | C | 10 | 22124263 | 437887 | ukb-b-5192 | 6.11E-05 | 48.15249 |
| rs4788616 | G | T | 16 | 72211984 | 437887 | ukb-b-5192 | 4.94E-05 | 38.98178 |
| rs4847408 | C | G | 1 | 93791437 | 437887 | ukb-b-5192 | 4.92E-05 | 39.06814 |
| rs494566 | T | C | 9 | 1785717 | 437887 | ukb-b-5192 | 4.58E-05 | 35.92984 |
| rs57555420 | T | C | 1 | 97783448 | 437887 | ukb-b-5192 | 4.19E-05 | 32.95692 |
| rs58541850 | A | G | 6 | 166165563 | 437887 | ukb-b-5192 | 5.04E-05 | 39.87179 |
| rs6102912 | C | T | 20 | 41202935 | 437887 | ukb-b-5192 | 5.76E-05 | 45.60277 |
| rs61743199 | G | A | 19 | 50161091 | 437887 | ukb-b-5192 | 4.03E-05 | 31.87378 |
| rs61864793 | C | T | 10 | 85803372 | 437887 | ukb-b-5192 | 4.68E-05 | 36.89429 |
| rs62145951 | C | T | 2 | 68399586 | 437887 | ukb-b-5192 | 5.61E-05 | 44.43287 |
| rs62199883 | A | C | 2 | 215376706 | 437887 | ukb-b-5192 | 9.57E-05 | 75.7684 |
| rs6511708 | C | T | 19 | 10788813 | 437887 | ukb-b-5192 | 6.51E-05 | 51.4104 |
| rs68056254 | T | G | 2 | 147846855 | 437887 | ukb-b-5192 | 4.67E-05 | 36.79187 |
| rs6814554 | A | G | 4 | 152454334 | 437887 | ukb-b-5192 | 9.13E-05 | 71.82265 |
| rs6850494 | C | A | 4 | 82291771 | 437887 | ukb-b-5192 | 4.42E-05 | 34.93805 |
| rs6895658 | C | T | 5 | 124274035 | 437887 | ukb-b-5192 | 5.25E-05 | 41.58331 |
| rs6994132 | C | T | 8 | 92653740 | 437887 | ukb-b-5192 | 4.84E-05 | 38.25 |
| rs7089973 | A | C | 10 | 116569565 | 437887 | ukb-b-5192 | 4.07E-05 | 31.89182 |
| rs71658797 | A | T | 1 | 77967507 | 437887 | ukb-b-5192 | 5.29E-05 | 41.69646 |
| rs72673939 | C | G | 8 | 118867693 | 437887 | ukb-b-5192 | 4.38E-05 | 34.55727 |
| rs73571431 | T | C | 9 | 126136139 | 437887 | ukb-b-5192 | 4.90E-05 | 38.49557 |
| rs73946726 | A | C | 2 | 117073427 | 437887 | ukb-b-5192 | 4.05E-05 | 31.34919 |
| rs749056 | G | T | 1 | 110037838 | 437887 | ukb-b-5192 | 4.25E-05 | 33.48163 |
| rs7539775 | A | G | 1 | 3109151 | 437887 | ukb-b-5192 | 3.79E-05 | 30.00513 |
| rs75499503 | T | C | 6 | 26145217 | 437887 | ukb-b-5192 | 0.000116 | 89.46348 |
| rs75641275 | C | A | 1 | 98327133 | 437887 | ukb-b-5192 | 5.85E-05 | 46.23892 |
| rs7708324 | G | A | 5 | 147920094 | 437887 | ukb-b-5192 | 4.59E-05 | 36.4041 |
| rs77273138 | A | T | 12 | 24092043 | 437887 | ukb-b-5192 | 4.78E-05 | 37.37918 |
| rs7798292 | A | G | 7 | 112974602 | 437887 | ukb-b-5192 | 4.64E-05 | 36.76417 |
| rs78227853 | T | C | 15 | 44169073 | 437887 | ukb-b-5192 | 3.89E-05 | 30.45264 |
| rs7899206 | G | T | 10 | 127188859 | 437887 | ukb-b-5192 | 4.78E-05 | 36.85235 |
| rs7921305 | A | G | 10 | 133775196 | 437887 | ukb-b-5192 | 4.97E-05 | 39.25695 |
| rs79373894 | C | T | 15 | 73369053 | 437887 | ukb-b-5192 | 5.48E-05 | 41.86217 |
| rs801733 | C | A | 11 | 65934549 | 437887 | ukb-b-5192 | 7.17E-05 | 56.72324 |
| rs814197 | G | T | 1 | 61092456 | 437887 | ukb-b-5192 | 5.68E-05 | 44.94817 |
| rs872169 | G | C | 2 | 24259188 | 437887 | ukb-b-5192 | 4.21E-05 | 33.23313 |
| rs883027 | C | G | 2 | 50600165 | 437887 | ukb-b-5192 | 4.56E-05 | 36.0163 |
| rs898751 | T | C | 17 | 2291863 | 437887 | ukb-b-5192 | 5.02E-05 | 39.66448 |
| rs9300594 | G | A | 13 | 100869905 | 437887 | ukb-b-5192 | 4.75E-05 | 37.50458 |
| rs9834970 | C | T | 3 | 36856030 | 437887 | ukb-b-5192 | 3.97E-05 | 31.49091 |
| rs9867437 | C | A | 3 | 85676752 | 437887 | ukb-b-5192 | 5.28E-05 | 41.36609 |
| rs9880023 | T | G | 3 | 54178199 | 437887 | ukb-b-5192 | 4.59E-05 | 35.99396 |
| rs996234 | A | G | 5 | 59455212 | 437887 | ukb-b-5192 | 5.55E-05 | 40.91593 |
| rs11191205 | A | G | 10 | 103831598 | 364465 | ukb-b-6811 | 3.29E-05 | 37.03415 |
| rs12956276 | A | G | 18 | 53175966 | 364465 | ukb-b-6811 | 3.26E-05 | 37.77121 |
| rs1368549 | T | G | 2 | 104064726 | 364465 | ukb-b-6811 | 4.18E-05 | 47.67754 |
| rs4580876 | A | G | 6 | 98322872 | 364465 | ukb-b-6811 | 6.73E-05 | 76.61328 |
| rs1028455 | A | T | 14 | 88829975 | 419314 | ukb-b-969 | 6.20E-05 | 33.22759 |
| rs10984444 | C | A | 9 | 121980347 | 419314 | ukb-b-969 | 6.90E-05 | 37.08904 |
| rs11776021 | A | G | 8 | 93025183 | 419314 | ukb-b-969 | 7.50E-05 | 40.40967 |
| rs12055997 | A | G | 7 | 11503851 | 419314 | ukb-b-969 | 5.92E-05 | 31.4534 |
| rs12203592 | T | C | 6 | 396321 | 419314 | ukb-b-969 | 6.97E-05 | 38.73921 |
| rs13251020 | C | T | 8 | 106257674 | 419314 | ukb-b-969 | 5.61E-05 | 30.03521 |
| rs1368551 | G | A | 2 | 104078658 | 419314 | ukb-b-969 | 0.000116 | 62.66337 |
| rs139577 | A | G | 22 | 47208945 | 419314 | ukb-b-969 | 7.15E-05 | 37.75577 |
| rs1449390 | A | G | 3 | 85577703 | 419314 | ukb-b-969 | 9.28E-05 | 50.04947 |
| rs145470583 | T | C | 7 | 71906931 | 419314 | ukb-b-969 | 5.99E-05 | 30.95418 |
| rs145748276 | A | G | 2 | 136485276 | 419314 | ukb-b-969 | 6.31E-05 | 34.34479 |
| rs2309849 | C | T | 2 | 101174309 | 419314 | ukb-b-969 | 5.54E-05 | 29.93137 |
| rs2356278 | C | T | 11 | 29022493 | 419314 | ukb-b-969 | 5.80E-05 | 31.29723 |
| rs2413639 | A | G | 22 | 41659580 | 419314 | ukb-b-969 | 6.12E-05 | 32.85339 |
| rs251033 | G | A | 16 | 72073122 | 419314 | ukb-b-969 | 8.51E-05 | 45.6866 |
| rs2647259 | A | C | 4 | 106256521 | 419314 | ukb-b-969 | 6.69E-05 | 36.03117 |
| rs34517439 | A | C | 1 | 78450517 | 419314 | ukb-b-969 | 8.96E-05 | 47.11832 |
| rs35660964 | A | G | 7 | 127272820 | 419314 | ukb-b-969 | 6.02E-05 | 32.20012 |
| rs35811586 | T | C | 2 | 233743794 | 419314 | ukb-b-969 | 5.53E-05 | 29.85581 |
| rs3849428 | T | C | 3 | 180802537 | 419314 | ukb-b-969 | 6.04E-05 | 32.75514 |
| rs4344697 | T | A | 15 | 82545214 | 419314 | ukb-b-969 | 7.53E-05 | 40.57266 |
| rs61083878 | G | A | 4 | 176956540 | 419314 | ukb-b-969 | 5.66E-05 | 30.28777 |
| rs644799 | G | A | 11 | 95564259 | 419314 | ukb-b-969 | 0.000104 | 56.27179 |
| rs7029718 | A | G | 9 | 23358495 | 419314 | ukb-b-969 | 7.40E-05 | 39.7834 |
| rs7191618 | G | C | 16 | 28565667 | 419314 | ukb-b-969 | 6.74E-05 | 36.29768 |
| rs7195043 | T | C | 16 | 90020861 | 419314 | ukb-b-969 | 6.79E-05 | 35.31413 |
| rs72673546 | T | C | 14 | 29834740 | 419314 | ukb-b-969 | 7.08E-05 | 38.11311 |
| rs7560588 | A | G | 2 | 60727416 | 419314 | ukb-b-969 | 6.22E-05 | 32.34841 |
| rs7578811 | G | T | 2 | 44834956 | 419314 | ukb-b-969 | 5.52E-05 | 29.81445 |
| rs7587930 | G | A | 2 | 199504423 | 419314 | ukb-b-969 | 9.67E-05 | 51.72129 |
| rs75900038 | A | G | 2 | 203412272 | 419314 | ukb-b-969 | 5.52E-05 | 29.76269 |
| rs7773004 | G | A | 6 | 26267755 | 419314 | ukb-b-969 | 8.77E-05 | 47.12604 |
| rs7852747 | G | A | 9 | 23688215 | 419314 | ukb-b-969 | 6.89E-05 | 37.0572 |
| rs837065 | T | C | 8 | 130853447 | 419314 | ukb-b-969 | 7.39E-05 | 39.56901 |
| rs9319835 | A | T | 18 | 69871763 | 419314 | ukb-b-969 | 5.98E-05 | 31.905 |
| rs9427232 | A | G | 1 | 153792958 | 419314 | ukb-b-969 | 5.70E-05 | 30.74499 |
| rs9508711 | A | G | 13 | 30912750 | 419314 | ukb-b-969 | 6.14E-05 | 32.99984 |
| rs9724773 | C | G | 1 | 98476966 | 419314 | ukb-b-969 | 8.34E-05 | 44.83759 |
| rs9852529 | A | G | 3 | 49730625 | 419314 | ukb-b-969 | 0.000116 | 62.54944 |

**Supplementary Table S2 All Mendelian randomization analysis results for entertainment activities on Alzheimer's disease in MR analysis.**

| Exposure | Outcome | Method | N SNP | OR | 95% LCI | 95% UCI | p-value |
| --- | --- | --- | --- | --- | --- | --- | --- |
| Leisure/social activities: Adult education class | Alzheimer’s Disease | Inverse variance weighted | 4 | 1.014044 | 0.985887 | 1.043004 | 0.331697 |
|  |  | MR Egger | 4 | 1.126799 | 0.932649 | 1.361365 | 0.341530 |
|  |  | Weighted median | 4 | 1.005345 | 0.973092 | 1.038667 | 0.748653 |
|  |  | Weighted mode | 4 | 1.003090 | 0.961411 | 1.046575 | 0.895737 |
| Leisure/social activities: Pub or social club | Alzheimer’s Disease | Inverse variance weighted | 15 | 1.004885 | 0.996950 | 1.012882 | 0.228278 |
|  |  | MR Egger | 15 | 1.010629 | 0.990344 | 1.031330 | 0.325388 |
|  |  | Weighted median | 15 | 1.006048 | 0.995023 | 1.017195 | 0.283456 |
|  |  | Weighted mode | 15 | 1.007706 | 0.993180 | 1.022445 | 0.317665 |
| Leisure/social activities: Religious group | Alzheimer’s Disease | Inverse variance weighted | 20 | 0.998389 | 0.988337 | 1.008543 | 0.754809 |
|  |  | MR Egger | 20 | 1.046285 | 0.967100 | 1.131953 | 0.274608 |
|  |  | Weighted median | 20 | 0.999050 | 0.986959 | 1.011289 | 0.878378 |
|  |  | Weighted mode | 20 | 0.997374 | 0.975219 | 1.020033 | 0.821018 |
| Leisure/social activities: Sports club or gym | Alzheimer’s Disease | Inverse variance weighted | 7 | 0.999378 | 0.986913 | 1.012000 | 0.922572 |
|  |  | MR Egger | 7 | 1.061758 | 0.942835 | 1.195681 | 0.368182 |
|  |  | Weighted median | 7 | 1.002951 | 0.985748 | 1.020454 | 0.738503 |
|  |  | Weighted mode | 7 | 1.011334 | 0.984604 | 1.038790 | 0.441099 |
| Leisure/social activities: Other group activity | Alzheimer’s Disease | Inverse variance weighted | 3 | 1.012375 | 0.978928 | 1.046966 | 0.473044 |
|  |  | MR Egger | 3 | 0.713628 | 0.506664 | 1.005135 | 0.304243 |
|  |  | Weighted median | 3 | 1.003792 | 0.972927 | 1.035635 | 0.812261 |
|  |  | Weighted mode | 3 | 0.998367 | 0.964999 | 1.032889 | 0.933528 |
| Leisure/social activities: None of the above | Alzheimer’s Disease | Inverse variance weighted | 10 | 0.999586 | 0.987684 | 1.011630 | 0.945914 |
|  |  | MR Egger | 10 | 0.982982 | 0.898282 | 1.075669 | 0.718585 |
|  |  | Weighted median | 10 | 1.001220 | 0.986723 | 1.015931 | 0.869836 |
|  |  | Weighted mode | 10 | 1.002536 | 0.980049 | 1.025539 | 0.831659 |
| Time spent doing light physical activity | Alzheimer’s Disease | Inverse variance weighted | 14 | 1.000779 | 0.998423 | 1.003141 | 0.517308 |
|  |  | MR Egger | 14 | 1.009521 | 0.980481 | 1.039422 | 0.536513 |
|  |  | Weighted median | 14 | 1.000850 | 0.997831 | 1.003878 | 0.581663 |
|  |  | Weighted mode | 14 | 1.004081 | 0.998174 | 1.010024 | 0.199150 |
| Time spent doing moderate physical activity | Alzheimer’s Disease | Inverse variance weighted | 15 | 0.999571 | 0.997995 | 1.001149 | 0.593830 |
|  |  | MR Egger | 15 | 1.001661 | 0.993130 | 1.010266 | 0.709838 |
|  |  | Weighted median | 15 | 0.999596 | 0.997505 | 1.001691 | 0.705089 |
|  |  | Weighted mode | 15 | 1.000388 | 0.997111 | 1.003675 | 0.820072 |
| Time spent doing vigorous physical activity | Alzheimer’s Disease | Inverse variance weighted | 9 | 0.999239 | 0.996913 | 1.001570 | 0.521981 |
|  |  | MR Egger | 9 | 0.999948 | 0.975924 | 1.024564 | 0.996793 |
|  |  | Weighted median | 9 | 0.999334 | 0.996265 | 1.002412 | 0.671141 |
|  |  | Weighted mode | 9 | 0.999375 | 0.994868 | 1.003903 | 0.793318 |
| Time spent driving | Alzheimer’s Disease | Inverse variance weighted | 6 | 1.000365 | 0.993520 | 1.007257 | 0.917079 |
|  |  | MR Egger | 6 | 1.022317 | 0.955600 | 1.093692 | 0.556389 |
|  |  | Weighted median | 6 | 0.997916 | 0.989478 | 1.006425 | 0.630063 |
|  |  | Weighted mode | 6 | 0.995359 | 0.983227 | 1.007641 | 0.490639 |
| Time spent outdoors in summer | Alzheimer’s Disease | Inverse variance weighted | 39 | 0.998394 | 0.995475 | 1.001322 | 0.281944 |
|  |  | MR Egger | 39 | 1.008532 | 0.993321 | 1.023975 | 0.280287 |
|  |  | Weighted median | 39 | 0.998868 | 0.995182 | 1.002567 | 0.547981 |
|  |  | Weighted mode | 39 | 0.999626 | 0.991374 | 1.007947 | 0.930026 |
| Time spent outdoors in winter | Alzheimer’s Disease | Inverse variance weighted | 4 | 0.992968 | 0.979925 | 1.006185 | 0.295542 |
|  |  | MR Egger | 4 | 1.062256 | 0.976664 | 1.155350 | 0.294179 |
|  |  | Weighted median | 4 | 0.993391 | 0.980469 | 1.006483 | 0.320866 |
|  |  | Weighted mode | 4 | 0.997635 | 0.980787 | 1.014773 | 0.802949 |
| Time spent using computer | Alzheimer’s Disease | Inverse variance weighted | 78 | 0.997719 | 0.995916 | 0.999524 | 0.013295 |
|  |  | MR Egger | 78 | 0.989500 | 0.980668 | 0.998411 | 0.023734 |
|  |  | Weighted median | 78 | 0.998419 | 0.995835 | 1.001009 | 0.231322 |
|  |  | Weighted mode | 78 | 0.999014 | 0.993022 | 1.005042 | 0.748813 |
| Time spent watching television (TV) | Alzheimer’s Disease | Inverse variance weighted | 103 | 1.000405 | 0.998619 | 1.002194 | 0.657091 |
|  |  | MR Egger | 103 | 1.005457 | 0.997628 | 1.013347 | 0.175420 |
|  |  | Weighted median | 103 | 1.000639 | 0.998015 | 1.003270 | 0.633448 |
|  |  | Weighted mode | 103 | 1.001281 | 0.994973 | 1.007630 | 0.692185 |

**Supplementary Table S3 Leave-one-out analyses for entertainment activities on Alzheimer's disease in MR analysis.**

| id.exposure | SNP | b | se | p-value |
| --- | --- | --- | --- | --- |
| ukb-b-151 | rs1491872 | -0.000672 | 0.001250 | 0.590761 |
| ukb-b-151 | rs2005617 | -0.000519 | 0.001251 | 0.678537 |
| ukb-b-151 | rs2189464 | -0.001015 | 0.001245 | 0.414756 |
| ukb-b-151 | rs2764261 | -0.000170 | 0.001268 | 0.893379 |
| ukb-b-151 | rs328900 | -0.000827 | 0.001268 | 0.514115 |
| ukb-b-151 | rs382210 | -0.000967 | 0.001266 | 0.445022 |
| ukb-b-151 | rs6955240 | -0.000394 | 0.001298 | 0.761395 |
| ukb-b-151 | rs7072776 | -0.001326 | 0.001252 | 0.289553 |
| ukb-b-151 | rs7749823 | -0.000925 | 0.001254 | 0.460545 |
| ukb-b-151 | All | -0.000761 | 0.001189 | 0.521981 |
| ukb-b-1553 | rs1125000 | 0.019984 | 0.016259 | 0.219026 |
| ukb-b-1553 | rs6997 | 0.019365 | 0.018835 | 0.303884 |
| ukb-b-1553 | rs72951028 | 0.001716 | 0.016387 | 0.916607 |
| ukb-b-1553 | rs9930477 | 0.015339 | 0.018156 | 0.398190 |
| ukb-b-1553 | All | 0.013946 | 0.014367 | 0.331697 |
| ukb-b-3793 | rs11057408 | 0.001900 | 0.003837 | 0.620564 |
| ukb-b-3793 | rs12069474 | 0.001201 | 0.004115 | 0.770455 |
| ukb-b-3793 | rs12921753 | 0.001811 | 0.004065 | 0.656004 |
| ukb-b-3793 | rs2090660 | -0.002480 | 0.003794 | 0.513344 |
| ukb-b-3793 | rs2588917 | -0.000090 | 0.004216 | 0.983012 |
| ukb-b-3793 | rs975303 | -0.000057 | 0.004310 | 0.989424 |
| ukb-b-3793 | All | 0.000365 | 0.003503 | 0.917079 |
| ukb-b-4000 | rs11712056 | 0.001415 | 0.007022 | 0.840326 |
| ukb-b-4000 | rs13002862 | -0.001083 | 0.007125 | 0.879142 |
| ukb-b-4000 | rs17315037 | -0.003014 | 0.006903 | 0.662329 |
| ukb-b-4000 | rs2499760 | -0.002531 | 0.006885 | 0.713218 |
| ukb-b-4000 | rs62263912 | -0.003998 | 0.007009 | 0.568404 |
| ukb-b-4000 | rs6478444 | 0.002376 | 0.006869 | 0.729385 |
| ukb-b-4000 | rs7228990 | 0.002396 | 0.006863 | 0.727055 |
| ukb-b-4000 | All | -0.000622 | 0.006403 | 0.922572 |
| ukb-b-4077 | rs11917431 | 0.015837 | 0.029567 | 0.592223 |
| ukb-b-4077 | rs17824247 | -0.003003 | 0.013006 | 0.817371 |
| ukb-b-4077 | rs2773485 | 0.024930 | 0.020528 | 0.224584 |
| ukb-b-4077 | All | 0.012300 | 0.017141 | 0.473044 |
| ukb-b-4171 | rs10978543 | 0.005266 | 0.004159 | 0.205461 |
| ukb-b-4171 | rs11066099 | 0.006321 | 0.004142 | 0.127024 |
| ukb-b-4171 | rs12103006 | 0.005145 | 0.004143 | 0.214330 |
| ukb-b-4171 | rs1229984 | 0.003263 | 0.004549 | 0.473206 |
| ukb-b-4171 | rs12472555 | 0.005616 | 0.004164 | 0.177464 |
| ukb-b-4171 | rs139920 | 0.006255 | 0.004198 | 0.136284 |
| ukb-b-4171 | rs2661863 | 0.003820 | 0.004148 | 0.357065 |
| ukb-b-4171 | rs35864506 | 0.004965 | 0.004163 | 0.233017 |
| ukb-b-4171 | rs36030660 | 0.004950 | 0.004155 | 0.233576 |
| ukb-b-4171 | rs56207132 | 0.003834 | 0.004144 | 0.354835 |
| ukb-b-4171 | rs61873510 | 0.004407 | 0.004160 | 0.289392 |
| ukb-b-4171 | rs6969458 | 0.004326 | 0.004257 | 0.309532 |
| ukb-b-4171 | rs754204 | 0.004048 | 0.004146 | 0.328820 |
| ukb-b-4171 | rs7545161 | 0.005639 | 0.004159 | 0.175103 |
| ukb-b-4171 | rs9401593 | 0.004980 | 0.004157 | 0.230956 |
| ukb-b-4171 | All | 0.004873 | 0.004044 | 0.228278 |
| ukb-b-4522 | rs10208088 | -0.002412 | 0.000927 | 0.009290 |
| ukb-b-4522 | rs1037091 | -0.002236 | 0.000932 | 0.016437 |
| ukb-b-4522 | rs10518019 | -0.002240 | 0.000927 | 0.015702 |
| ukb-b-4522 | rs11259902 | -0.002306 | 0.000927 | 0.012907 |
| ukb-b-4522 | rs112600282 | -0.002127 | 0.000928 | 0.021871 |
| ukb-b-4522 | rs113851275 | -0.002333 | 0.000929 | 0.012016 |
| ukb-b-4522 | rs11634155 | -0.002484 | 0.000928 | 0.007440 |
| ukb-b-4522 | rs11652437 | -0.002208 | 0.000929 | 0.017459 |
| ukb-b-4522 | rs117405403 | -0.002272 | 0.000929 | 0.014425 |
| ukb-b-4522 | rs11749912 | -0.002249 | 0.000930 | 0.015589 |
| ukb-b-4522 | rs11766392 | -0.002233 | 0.000929 | 0.016173 |
| ukb-b-4522 | rs11942953 | -0.002184 | 0.000928 | 0.018527 |
| ukb-b-4522 | rs12128707 | -0.002452 | 0.000928 | 0.008222 |
| ukb-b-4522 | rs12145677 | -0.002358 | 0.000932 | 0.011412 |
| ukb-b-4522 | rs1229984 | -0.002185 | 0.000928 | 0.018579 |
| ukb-b-4522 | rs12521638 | -0.002291 | 0.000927 | 0.013478 |
| ukb-b-4522 | rs12553324 | -0.001882 | 0.000936 | 0.044478 |
| ukb-b-4522 | rs12706626 | -0.002408 | 0.000927 | 0.009420 |
| ukb-b-4522 | rs12820967 | -0.002355 | 0.000928 | 0.011163 |
| ukb-b-4522 | rs12946454 | -0.002309 | 0.000928 | 0.012852 |
| ukb-b-4522 | rs13262595 | -0.002368 | 0.000934 | 0.011226 |
| ukb-b-4522 | rs13422733 | -0.002307 | 0.000927 | 0.012856 |
| ukb-b-4522 | rs136553 | -0.002357 | 0.000928 | 0.011099 |
| ukb-b-4522 | rs1395020 | -0.002335 | 0.000927 | 0.011802 |
| ukb-b-4522 | rs1448355 | -0.002390 | 0.000929 | 0.010100 |
| ukb-b-4522 | rs1469249 | -0.002214 | 0.000928 | 0.017025 |
| ukb-b-4522 | rs147543875 | -0.002256 | 0.000927 | 0.014995 |
| ukb-b-4522 | rs1648906 | -0.002232 | 0.000927 | 0.016095 |
| ukb-b-4522 | rs166835 | -0.002398 | 0.000928 | 0.009759 |
| ukb-b-4522 | rs16912540 | -0.002305 | 0.000928 | 0.013004 |
| ukb-b-4522 | rs17167210 | -0.002265 | 0.000928 | 0.014711 |
| ukb-b-4522 | rs17789218 | -0.002246 | 0.000928 | 0.015537 |
| ukb-b-4522 | rs17862355 | -0.002368 | 0.000928 | 0.010756 |
| ukb-b-4522 | rs1987942 | -0.002202 | 0.000928 | 0.017602 |
| ukb-b-4522 | rs2032780 | -0.002483 | 0.000929 | 0.007541 |
| ukb-b-4522 | rs2068625 | -0.002254 | 0.000930 | 0.015407 |
| ukb-b-4522 | rs206965 | -0.002167 | 0.000928 | 0.019497 |
| ukb-b-4522 | rs2120461 | -0.002175 | 0.000929 | 0.019198 |
| ukb-b-4522 | rs2220599 | -0.002143 | 0.000929 | 0.021072 |
| ukb-b-4522 | rs246723 | -0.002526 | 0.000928 | 0.006455 |
| ukb-b-4522 | rs2588543 | -0.002321 | 0.000927 | 0.012309 |
| ukb-b-4522 | rs2734833 | -0.002429 | 0.000929 | 0.008949 |
| ukb-b-4522 | rs2748985 | -0.002393 | 0.000930 | 0.010053 |
| ukb-b-4522 | rs2756121 | -0.002256 | 0.000928 | 0.015092 |
| ukb-b-4522 | rs2761438 | -0.002127 | 0.000928 | 0.021914 |
| ukb-b-4522 | rs28710456 | -0.002292 | 0.000928 | 0.013471 |
| ukb-b-4522 | rs306755 | -0.002355 | 0.000928 | 0.011125 |
| ukb-b-4522 | rs34238696 | -0.002044 | 0.000928 | 0.027566 |
| ukb-b-4522 | rs3730399 | -0.002375 | 0.000928 | 0.010508 |
| ukb-b-4522 | rs4704043 | -0.002233 | 0.000928 | 0.016060 |
| ukb-b-4522 | rs4852252 | -0.002253 | 0.000927 | 0.015118 |
| ukb-b-4522 | rs56229818 | -0.002427 | 0.000927 | 0.008861 |
| ukb-b-4522 | rs6028090 | -0.002275 | 0.000930 | 0.014398 |
| ukb-b-4522 | rs613872 | -0.002301 | 0.000929 | 0.013260 |
| ukb-b-4522 | rs6449708 | -0.002281 | 0.000928 | 0.013959 |
| ukb-b-4522 | rs6780848 | -0.002349 | 0.000927 | 0.011298 |
| ukb-b-4522 | rs6935828 | -0.002305 | 0.000927 | 0.012909 |
| ukb-b-4522 | rs7020477 | -0.002301 | 0.000928 | 0.013141 |
| ukb-b-4522 | rs707926 | -0.002202 | 0.000928 | 0.017612 |
| ukb-b-4522 | rs7209653 | -0.002219 | 0.000929 | 0.016968 |
| ukb-b-4522 | rs7281293 | -0.002384 | 0.000928 | 0.010206 |
| ukb-b-4522 | rs72828532 | -0.002313 | 0.000929 | 0.012807 |
| ukb-b-4522 | rs72847500 | -0.002311 | 0.000927 | 0.012693 |
| ukb-b-4522 | rs7288455 | -0.002282 | 0.000928 | 0.013891 |
| ukb-b-4522 | rs73578186 | -0.002293 | 0.000929 | 0.013531 |
| ukb-b-4522 | rs7526112 | -0.002208 | 0.000928 | 0.017291 |
| ukb-b-4522 | rs75550998 | -0.002312 | 0.000927 | 0.012656 |
| ukb-b-4522 | rs7564844 | -0.002435 | 0.000928 | 0.008736 |
| ukb-b-4522 | rs76112266 | -0.002135 | 0.000929 | 0.021565 |
| ukb-b-4522 | rs7630869 | -0.002346 | 0.000933 | 0.011921 |
| ukb-b-4522 | rs76824303 | -0.002349 | 0.000929 | 0.011480 |
| ukb-b-4522 | rs7904398 | -0.002190 | 0.000927 | 0.018215 |
| ukb-b-4522 | rs7968738 | -0.002169 | 0.000928 | 0.019441 |
| ukb-b-4522 | rs79720045 | -0.002171 | 0.000930 | 0.019485 |
| ukb-b-4522 | rs806795 | -0.002256 | 0.000927 | 0.015009 |
| ukb-b-4522 | rs8102851 | -0.002209 | 0.000928 | 0.017264 |
| ukb-b-4522 | rs9375188 | -0.002318 | 0.000934 | 0.013087 |
| ukb-b-4522 | rs9537571 | -0.002220 | 0.000928 | 0.016797 |
| ukb-b-4522 | All | -0.002284 | 0.000923 | 0.013295 |
| ukb-b-4667 | rs11877152 | -0.001162 | 0.005405 | 0.829749 |
| ukb-b-4667 | rs12119422 | -0.002496 | 0.005316 | 0.638754 |
| ukb-b-4667 | rs12156017 | -0.000376 | 0.005290 | 0.943388 |
| ukb-b-4667 | rs12992090 | -0.001768 | 0.005467 | 0.746402 |
| ukb-b-4667 | rs1471093 | -0.001363 | 0.005494 | 0.803984 |
| ukb-b-4667 | rs17527878 | -0.001380 | 0.005428 | 0.799333 |
| ukb-b-4667 | rs1991083 | -0.003350 | 0.005085 | 0.510016 |
| ukb-b-4667 | rs3197999 | -0.002201 | 0.005389 | 0.682912 |
| ukb-b-4667 | rs332828 | -0.002166 | 0.005465 | 0.691886 |
| ukb-b-4667 | rs34402524 | -0.003000 | 0.005256 | 0.568110 |
| ukb-b-4667 | rs36104984 | -0.002222 | 0.005377 | 0.679432 |
| ukb-b-4667 | rs410671 | -0.000931 | 0.005444 | 0.864167 |
| ukb-b-4667 | rs6545977 | -0.001708 | 0.005417 | 0.752472 |
| ukb-b-4667 | rs6722794 | -0.000676 | 0.005316 | 0.898751 |
| ukb-b-4667 | rs6862251 | -0.001454 | 0.005453 | 0.789779 |
| ukb-b-4667 | rs6944796 | -0.003057 | 0.005261 | 0.561258 |
| ukb-b-4667 | rs699534 | -0.003082 | 0.005239 | 0.556379 |
| ukb-b-4667 | rs7388625 | 0.000652 | 0.004721 | 0.890137 |
| ukb-b-4667 | rs8020432 | -0.001456 | 0.005410 | 0.787753 |
| ukb-b-4667 | rs990702 | 0.000915 | 0.004725 | 0.846476 |
| ukb-b-4667 | All | -0.001612 | 0.005163 | 0.754809 |
| ukb-b-4710 | rs10098073 | -0.000436 | 0.000862 | 0.612926 |
| ukb-b-4710 | rs11749912 | -0.000583 | 0.000858 | 0.496516 |
| ukb-b-4710 | rs11913445 | -0.000411 | 0.000855 | 0.631107 |
| ukb-b-4710 | rs2246122 | -0.000309 | 0.000857 | 0.718519 |
| ukb-b-4710 | rs3094622 | -0.000542 | 0.000877 | 0.536476 |
| ukb-b-4710 | rs3129962 | -0.000502 | 0.000861 | 0.559834 |
| ukb-b-4710 | rs34775997 | -0.000398 | 0.000856 | 0.641997 |
| ukb-b-4710 | rs4129572 | -0.000232 | 0.000872 | 0.790476 |
| ukb-b-4710 | rs4540651 | -0.000224 | 0.000857 | 0.793441 |
| ukb-b-4710 | rs4886868 | -0.000213 | 0.000860 | 0.804326 |
| ukb-b-4710 | rs7229874 | -0.000581 | 0.000856 | 0.497529 |
| ukb-b-4710 | rs7565480 | -0.000591 | 0.000856 | 0.490495 |
| ukb-b-4710 | rs9533455 | -0.000256 | 0.000858 | 0.765495 |
| ukb-b-4710 | rs997467 | -0.000553 | 0.000862 | 0.521114 |
| ukb-b-4710 | All | -0.000416 | 0.000829 | 0.615483 |
| ukb-b-4886 | rs11183184 | 0.000900 | 0.001286 | 0.484266 |
| ukb-b-4886 | rs12568280 | 0.001111 | 0.001248 | 0.373121 |
| ukb-b-4886 | rs2011071 | 0.000556 | 0.001268 | 0.661043 |
| ukb-b-4886 | rs2220599 | 0.000318 | 0.001243 | 0.797878 |
| ukb-b-4886 | rs34654885 | 0.000928 | 0.001295 | 0.473706 |
| ukb-b-4886 | rs4588066 | 0.000352 | 0.001194 | 0.768031 |
| ukb-b-4886 | rs4641022 | 0.000497 | 0.001250 | 0.691224 |
| ukb-b-4886 | rs62172117 | 0.001485 | 0.001104 | 0.178787 |
| ukb-b-4886 | rs62422661 | 0.000889 | 0.001310 | 0.497638 |
| ukb-b-4886 | rs62482241 | 0.000438 | 0.001245 | 0.724817 |
| ukb-b-4886 | rs72931435 | 0.000602 | 0.001274 | 0.636409 |
| ukb-b-4886 | rs747344 | 0.001223 | 0.001178 | 0.299164 |
| ukb-b-4886 | rs7576657 | 0.000623 | 0.001295 | 0.630444 |
| ukb-b-4886 | rs9400239 | 0.000984 | 0.001268 | 0.437642 |
| ukb-b-4886 | All | 0.000779 | 0.001203 | 0.517308 |
| ukb-b-5076 | rs12889064 | 0.002659 | 0.005732 | 0.642797 |
| ukb-b-5076 | rs13011181 | -0.000329 | 0.006987 | 0.962485 |
| ukb-b-5076 | rs2043145 | 0.001542 | 0.006457 | 0.811239 |
| ukb-b-5076 | rs2675638 | -0.002281 | 0.006428 | 0.722698 |
| ukb-b-5076 | rs3197999 | 0.000550 | 0.006843 | 0.935893 |
| ukb-b-5076 | rs35291206 | -0.001041 | 0.006744 | 0.877356 |
| ukb-b-5076 | rs410671 | -0.001532 | 0.006659 | 0.818092 |
| ukb-b-5076 | rs4247450 | -0.003996 | 0.005425 | 0.461446 |
| ukb-b-5076 | rs4470910 | -0.001278 | 0.006805 | 0.851001 |
| ukb-b-5076 | rs847687 | 0.001594 | 0.006350 | 0.801781 |
| ukb-b-5076 | All | -0.000415 | 0.006111 | 0.945914 |
| ukb-b-5192 | rs10109061 | 0.000510 | 0.000924 | 0.581176 |
| ukb-b-5192 | rs10189857 | 0.000437 | 0.000929 | 0.638114 |
| ukb-b-5192 | rs10269099 | 0.000519 | 0.000924 | 0.574048 |
| ukb-b-5192 | rs10739499 | 0.000527 | 0.000923 | 0.568255 |
| ukb-b-5192 | rs10765777 | 0.000493 | 0.000926 | 0.594514 |
| ukb-b-5192 | rs11191129 | 0.000532 | 0.000924 | 0.565124 |
| ukb-b-5192 | rs11222919 | 0.000517 | 0.000924 | 0.575647 |
| ukb-b-5192 | rs11245482 | 0.000573 | 0.000922 | 0.534204 |
| ukb-b-5192 | rs114600294 | 0.000466 | 0.000923 | 0.613190 |
| ukb-b-5192 | rs114755463 | 0.000414 | 0.000920 | 0.652324 |
| ukb-b-5192 | rs115608101 | 0.000513 | 0.000924 | 0.579014 |
| ukb-b-5192 | rs11662211 | 0.000411 | 0.000919 | 0.654760 |
| ukb-b-5192 | rs11680095 | 0.000633 | 0.000912 | 0.487361 |
| ukb-b-5192 | rs11696187 | 0.000405 | 0.000919 | 0.659334 |
| ukb-b-5192 | rs11700249 | 0.000491 | 0.000924 | 0.594918 |
| ukb-b-5192 | rs11714337 | 0.000370 | 0.000914 | 0.685272 |
| ukb-b-5192 | rs11877758 | 0.000389 | 0.000918 | 0.672012 |
| ukb-b-5192 | rs11911112 | 0.000513 | 0.000925 | 0.579516 |
| ukb-b-5192 | rs12045585 | 0.000337 | 0.000908 | 0.710618 |
| ukb-b-5192 | rs12214364 | 0.000511 | 0.000924 | 0.580524 |
| ukb-b-5192 | rs12553324 | 0.000171 | 0.000906 | 0.850773 |
| ukb-b-5192 | rs1291871 | 0.000555 | 0.000922 | 0.547639 |
| ukb-b-5192 | rs13014947 | 0.000676 | 0.000909 | 0.456955 |
| ukb-b-5192 | rs13107325 | 0.000581 | 0.000923 | 0.528508 |
| ukb-b-5192 | rs1324491 | 0.000663 | 0.000906 | 0.464183 |
| ukb-b-5192 | rs1727332 | 0.000472 | 0.000926 | 0.610441 |
| ukb-b-5192 | rs17789218 | 0.000455 | 0.000923 | 0.621619 |
| ukb-b-5192 | rs180396 | 0.000491 | 0.000924 | 0.595033 |
| ukb-b-5192 | rs1826510 | 0.000452 | 0.000922 | 0.624002 |
| ukb-b-5192 | rs184332798 | 0.000604 | 0.000917 | 0.509826 |
| ukb-b-5192 | rs1993092 | 0.000447 | 0.000922 | 0.628126 |
| ukb-b-5192 | rs2073869 | 0.000610 | 0.000919 | 0.507191 |
| ukb-b-5192 | rs2106164 | 0.000555 | 0.000923 | 0.547395 |
| ukb-b-5192 | rs2185490 | 0.000476 | 0.000924 | 0.605899 |
| ukb-b-5192 | rs2240857 | 0.000530 | 0.000925 | 0.566964 |
| ukb-b-5192 | rs2283 | 0.000309 | 0.000902 | 0.732169 |
| ukb-b-5192 | rs2332818 | 0.000522 | 0.000924 | 0.571925 |
| ukb-b-5192 | rs2352984 | 0.000398 | 0.000936 | 0.670760 |
| ukb-b-5192 | rs2479968 | 0.000559 | 0.000922 | 0.543996 |
| ukb-b-5192 | rs249960 | 0.000563 | 0.000922 | 0.541630 |
| ukb-b-5192 | rs262890 | 0.000500 | 0.000927 | 0.589720 |
| ukb-b-5192 | rs263771 | 0.000601 | 0.000920 | 0.513149 |
| ukb-b-5192 | rs2646351 | 0.000517 | 0.000924 | 0.575901 |
| ukb-b-5192 | rs2678662 | 0.000713 | 0.000904 | 0.430696 |
| ukb-b-5192 | rs2725371 | 0.000388 | 0.000922 | 0.673931 |
| ukb-b-5192 | rs2857693 | 0.000495 | 0.000925 | 0.592467 |
| ukb-b-5192 | rs2906604 | 0.000412 | 0.000921 | 0.654222 |
| ukb-b-5192 | rs3138499 | 0.000439 | 0.000924 | 0.634543 |
| ukb-b-5192 | rs34811474 | 0.000441 | 0.000924 | 0.633277 |
| ukb-b-5192 | rs35797019 | 0.000466 | 0.000923 | 0.613414 |
| ukb-b-5192 | rs362312 | 0.000595 | 0.000919 | 0.517570 |
| ukb-b-5192 | rs3754970 | 0.000552 | 0.000923 | 0.549645 |
| ukb-b-5192 | rs3810496 | 0.000519 | 0.000924 | 0.574602 |
| ukb-b-5192 | rs4076457 | 0.000407 | 0.000917 | 0.656709 |
| ukb-b-5192 | rs4110177 | 0.000476 | 0.000923 | 0.606028 |
| ukb-b-5192 | rs4303732 | 0.000509 | 0.000925 | 0.582233 |
| ukb-b-5192 | rs4339469 | 0.000589 | 0.000924 | 0.524224 |
| ukb-b-5192 | rs4469687 | 0.000489 | 0.000924 | 0.596416 |
| ukb-b-5192 | rs4567133 | 0.000496 | 0.000925 | 0.591945 |
| ukb-b-5192 | rs4747438 | 0.000628 | 0.000919 | 0.494022 |
| ukb-b-5192 | rs4788616 | 0.000532 | 0.000924 | 0.564764 |
| ukb-b-5192 | rs4847408 | 0.000403 | 0.000919 | 0.661097 |
| ukb-b-5192 | rs494566 | 0.000601 | 0.000919 | 0.512621 |
| ukb-b-5192 | rs57555420 | 0.000567 | 0.000921 | 0.538235 |
| ukb-b-5192 | rs58541850 | 0.000444 | 0.000923 | 0.630431 |
| ukb-b-5192 | rs6102912 | 0.000586 | 0.000922 | 0.525123 |
| ukb-b-5192 | rs61743199 | 0.000376 | 0.000912 | 0.680292 |
| ukb-b-5192 | rs61864793 | 0.000504 | 0.000925 | 0.585620 |
| ukb-b-5192 | rs62145951 | 0.000332 | 0.000910 | 0.715554 |
| ukb-b-5192 | rs62199883 | 0.000542 | 0.000928 | 0.559558 |
| ukb-b-5192 | rs6511708 | 0.000635 | 0.000919 | 0.489147 |
| ukb-b-5192 | rs68056254 | 0.000513 | 0.000924 | 0.579176 |
| ukb-b-5192 | rs6814554 | 0.000467 | 0.000928 | 0.614839 |
| ukb-b-5192 | rs6850494 | 0.000460 | 0.000923 | 0.617862 |
| ukb-b-5192 | rs6895658 | 0.000386 | 0.000917 | 0.673573 |
| ukb-b-5192 | rs6994132 | 0.000509 | 0.000925 | 0.581819 |
| ukb-b-5192 | rs7089973 | 0.000481 | 0.000924 | 0.602234 |
| ukb-b-5192 | rs71658797 | 0.000519 | 0.000925 | 0.574992 |
| ukb-b-5192 | rs72673939 | 0.000421 | 0.000920 | 0.647002 |
| ukb-b-5192 | rs73571431 | 0.000412 | 0.000920 | 0.654435 |
| ukb-b-5192 | rs73946726 | 0.000419 | 0.000919 | 0.647943 |
| ukb-b-5192 | rs749056 | 0.000529 | 0.000924 | 0.566889 |
| ukb-b-5192 | rs7539775 | 0.000559 | 0.000922 | 0.544292 |
| ukb-b-5192 | rs75499503 | 0.000591 | 0.000929 | 0.524460 |
| ukb-b-5192 | rs75641275 | 0.000591 | 0.000922 | 0.521616 |
| ukb-b-5192 | rs7708324 | 0.000622 | 0.000916 | 0.497439 |
| ukb-b-5192 | rs77273138 | 0.000422 | 0.000920 | 0.646670 |
| ukb-b-5192 | rs7798292 | 0.000583 | 0.000921 | 0.526932 |
| ukb-b-5192 | rs78227853 | 0.000518 | 0.000924 | 0.574824 |
| ukb-b-5192 | rs7899206 | 0.000559 | 0.000923 | 0.544786 |
| ukb-b-5192 | rs7921305 | 0.000611 | 0.000918 | 0.506041 |
| ukb-b-5192 | rs79373894 | 0.000478 | 0.000925 | 0.605069 |
| ukb-b-5192 | rs801733 | 0.000686 | 0.000914 | 0.452930 |
| ukb-b-5192 | rs814197 | 0.000388 | 0.000919 | 0.672676 |
| ukb-b-5192 | rs872169 | 0.000607 | 0.000917 | 0.508099 |
| ukb-b-5192 | rs883027 | 0.000676 | 0.000906 | 0.455675 |
| ukb-b-5192 | rs898751 | 0.000476 | 0.000924 | 0.606872 |
| ukb-b-5192 | rs9300594 | 0.000501 | 0.000925 | 0.588004 |
| ukb-b-5192 | rs9834970 | 0.000730 | 0.000902 | 0.418285 |
| ukb-b-5192 | rs9867437 | 0.000459 | 0.000924 | 0.619656 |
| ukb-b-5192 | rs9880023 | 0.000457 | 0.000923 | 0.620514 |
| ukb-b-5192 | rs996234 | 0.000446 | 0.000923 | 0.628697 |
| ukb-b-5192 | All | 0.000505 | 0.000916 | 0.581588 |
| ukb-b-6811 | rs11191205 | -0.010844 | 0.006724 | 0.106839 |
| ukb-b-6811 | rs12956276 | -0.005078 | 0.008597 | 0.554759 |
| ukb-b-6811 | rs1368549 | -0.002136 | 0.006262 | 0.732970 |
| ukb-b-6811 | rs4580876 | -0.010736 | 0.009668 | 0.266796 |
| ukb-b-6811 | All | -0.007057 | 0.006746 | 0.295542 |
| ukb-b-969 | rs1028455 | -0.001419 | 0.001517 | 0.349497 |
| ukb-b-969 | rs10984444 | -0.001278 | 0.001494 | 0.392486 |
| ukb-b-969 | rs11776021 | -0.001669 | 0.001534 | 0.276550 |
| ukb-b-969 | rs12055997 | -0.001467 | 0.001522 | 0.335115 |
| ukb-b-969 | rs12203592 | -0.001275 | 0.001491 | 0.392656 |
| ukb-b-969 | rs13251020 | -0.001492 | 0.001524 | 0.327358 |
| ukb-b-969 | rs1368551 | -0.001136 | 0.001500 | 0.448950 |
| ukb-b-969 | rs139577 | -0.001884 | 0.001507 | 0.211125 |
| ukb-b-969 | rs1449390 | -0.001870 | 0.001522 | 0.219151 |
| ukb-b-969 | rs145470583 | -0.001594 | 0.001530 | 0.297409 |
| ukb-b-969 | rs145748276 | -0.002072 | 0.001443 | 0.151071 |
| ukb-b-969 | rs2309849 | -0.001367 | 0.001504 | 0.363310 |
| ukb-b-969 | rs2356278 | -0.001803 | 0.001514 | 0.233647 |
| ukb-b-969 | rs2413639 | -0.001626 | 0.001531 | 0.288227 |
| ukb-b-969 | rs251033 | -0.001626 | 0.001538 | 0.290433 |
| ukb-b-969 | rs2647259 | -0.001187 | 0.001467 | 0.418408 |
| ukb-b-969 | rs34517439 | -0.001845 | 0.001523 | 0.225682 |
| ukb-b-969 | rs35660964 | -0.001618 | 0.001531 | 0.290575 |
| ukb-b-969 | rs35811586 | -0.001750 | 0.001520 | 0.249769 |
| ukb-b-969 | rs3849428 | -0.001742 | 0.001524 | 0.253007 |
| ukb-b-969 | rs4344697 | -0.001611 | 0.001535 | 0.293966 |
| ukb-b-969 | rs61083878 | -0.001542 | 0.001528 | 0.312980 |
| ukb-b-969 | rs644799 | -0.001697 | 0.001542 | 0.270872 |
| ukb-b-969 | rs7029718 | -0.002264 | 0.001386 | 0.102225 |
| ukb-b-969 | rs7191618 | -0.001261 | 0.001489 | 0.396952 |
| ukb-b-969 | rs7195043 | -0.001703 | 0.001529 | 0.265327 |
| ukb-b-969 | rs72673546 | -0.001925 | 0.001499 | 0.199034 |
| ukb-b-969 | rs7560588 | -0.001888 | 0.001499 | 0.207812 |
| ukb-b-969 | rs7578811 | -0.001728 | 0.001523 | 0.256617 |
| ukb-b-969 | rs7587930 | -0.001469 | 0.001536 | 0.339086 |
| ukb-b-969 | rs75900038 | -0.001664 | 0.001528 | 0.276141 |
| ukb-b-969 | rs7773004 | -0.001725 | 0.001535 | 0.260960 |
| ukb-b-969 | rs7852747 | -0.001825 | 0.001516 | 0.228719 |
| ukb-b-969 | rs837065 | -0.001527 | 0.001532 | 0.318990 |
| ukb-b-969 | rs9319835 | -0.001436 | 0.001518 | 0.344399 |
| ukb-b-969 | rs9427232 | -0.001393 | 0.001510 | 0.356316 |
| ukb-b-969 | rs9508711 | -0.001333 | 0.001501 | 0.374337 |
| ukb-b-969 | rs9724773 | -0.001451 | 0.001530 | 0.343061 |
| ukb-b-969 | rs9852529 | -0.001526 | 0.001545 | 0.323562 |
| ukb-b-969 | All | -0.001607 | 0.001494 | 0.281944 |
